# From Chemical Transformations to Cup Quality: Integrated Insights into Key Non-Volatile Compounds, Aroma-Active Compounds and Sensory Evolution During Continuous Roasting Stages in Yunnan Arabica Coffee

**DOI:** 10.3390/foods15193569

**Published:** 2026-10-08

**Authors:** Yizhi Chen, Siqian Guo, Yilin Wang, Xianwen Wang, Tao Tong, Wentao Gao, Kexi Ma, Liyan Ma

**Affiliations:** 1 College of Food Science & Nutritional Engineering, China Agricultural University, No. 17 Tsinghua Dong Road, Beijing 100083, China; chenyizhi10200820@163.com (Y.C.); guosiqian2022@163.com (S.G.); 15887170929@163.com (Y.W.); tongtao1028@cau.edu.cn (T.T.); gaowentao636@163.com (W.G.); makexi2009@163.com (K.M.); 2 CAU Sichuan Chengdu Advanced Agricultural Industrial Institute, No. 515 Xingyuan 8th Road, Chengdu 611430, China; 3 Baoshan Inspection and Testing Institute, Baoshan 678000, China; awenchina@163.com; 4 Key Laboratory of Coffee Quality and Safety, State Administration for Market Regulation, Baoshan 678000, China; 5 Inspection and Testing Center for Agricultural Products Quality, Ministry of Agriculture and Rural Affairs, Beijing 100083, China

**Keywords:** Arabica coffee, roasting stage, aroma-active compound, aroma, cup quality

## Abstract

Although understanding aroma evolution during roasting is essential for optimizing roasting control and coffee quality, most studies have focused on only a few discrete roasting levels. In this study, high-performance liquid chromatography (HPLC), headspace solid-phase microextraction and gas chromatography–mass spectrometry (HS-SPME-GC–MS), and sensory evaluation were integrated to characterize changes in key non-volatile compounds, odor-active compounds, and cup quality of Arabica coffee from green beans (S0) through eight successive roasting stages (S1–S8). The results show that the total contents of soluble sugars and chlorogenic acids continuously decreased throughout the roasting process. Volatile compound (VOC) diversity increased until S5, while the total VOC concentration peaked at S7. A total of 47 VOCs showed OAVs > 1, and phenylethyl alcohol, furfuryl alcohol, furfural, phenol, and pyridine were identified as key discriminant markers. Cup quality improved progressively and reached its optimum at S4, characterized by balanced nutty, roasted hazelnut-like, and caramel-like notes, but declined thereafter as smoky and burnt–bitter characteristics intensified. The transition from S4 to S5 therefore represents a critical turning point in coffee aroma evolution during roasting. These findings provide a basis for optimizing roasting control and coffee aroma quality.

## 1. Introduction

Coffee is one of the most popular beverages worldwide [1], which is appreciated for its potential health-promoting properties, stimulating effects, and distinctive sensory characteristics, particularly its complex aroma [2,3,4]. Originating in Ethiopia, coffee is a tropical crop of the Rubiaceae family that is now cultivated extensively throughout Central and South America, Africa, and Asia [5,6]. With the continuous growth in global coffee demand, the coffee industry in China has also developed rapidly [7]. Yunnan Province is the most important coffee-producing region in China, where Arabica coffee accounts for more than 98% of the total local coffee-growing area and production [8]. Among the major coffee-producing areas in Yunnan, Baoshan has a long history of coffee cultivation and is recognized as one of the representative origins of Yunnan small-grain coffee. Arabica coffee produced in Baoshan exhibits a distinctive flavor profile, commonly characterized as “fragrant but not overpowering, full-bodied but not bitter, with subtle fruity notes” [9]. Among the sensory attributes determining coffee quality, aroma is an important component of overall flavor and plays a critical role in determining consumer perception and cup quality [10].

Coffee aroma is influenced by multiple factors, including geographical origin, cultivar, processing method, roasting degree, and brewing conditions [11,12,13,14,15,16]. Among these factors, roasting degree plays a particularly important role in shaping the final aroma profile. Unroasted green coffee beans generally lack the pleasant and characteristic aroma associated with roasted coffee [17]. During roasting, a series of thermally induced reactions drive the formation, transformation, and degradation of aroma-related compounds, gradually converting green coffee beans into roasted coffee with a complex aroma profile [18]. In recent years, the relationship between roasting degree and coffee aroma has been extensively investigated. By monitoring the continuous roasting process of Arabica and C. canephora coffees, Debona et al. (2025) found that coffee exhibited greater volatile diversity and relatively higher sensory quality at the medium-roasting stage [19]. In Yunnan Arabica coffee, He et al. have reported that beany, grassy, and cereal-like notes gradually weakened with increasing roasting degree, whereas caramel-like, nutty, and roasted notes became more pronounced [20]. Yuan et al. (2025) further demonstrated that the profiles of odor-active compounds differed markedly among roasting degrees and that their formation was closely associated with lipid oxidation, phenolic acid degradation, Maillard reactions, and caramelization [21]. Ban et al. (2025) further showed that roasting degree affected volatile formation and taste characteristics, while caffeine was only weakly associated with bitterness [22]. Overall, the volatile composition and sensory characteristics of coffee continuously evolve as roasting progresses. This process is fundamentally linked to the ongoing transformation of various non-volatile aroma precursors [23]. Among the non-volatile constituents undergoing pronounced changes during roasting, soluble sugars and chlorogenic acids are particularly relevant to the development of coffee flavor. Soluble sugars serve as important substrates for Maillard and caramelization reactions, thereby contributing to the formation of volatile compounds (VOCs) associated with caramel-like and roasted aroma [24]. Chlorogenic acids not only contribute directly to coffee taste but also undergo extensive isomerization and thermal degradation during roasting, with their transformation products further influencing the development of coffee flavor [25]. Caffeine is another important non-volatile component of coffee that contributes to its quality and physiological stimulant properties [26].

Chemical profiling approaches, including high-performance liquid chromatography (HPLC) and headspace solid-phase microextraction coupled with gas chromatography–mass spectrometry (HS-SPME-GC-MS), have been widely applied to characterize non-volatile and volatile compounds in coffee, respectively [27,28]. However, instrumental analysis alone cannot fully explain sensory differences in coffee quality, while sensory evaluation provides direct information on perceived quality but limited insight into the chemical basis underlying these differences [9]. Therefore, integrating chemical analysis with sensory evaluation provides a more comprehensive approach for linking molecular changes with sensory consequences during coffee roasting [29,30]. Recent studies have provided important insights into the evolution of coffee aroma during roasting through comprehensive characterization of VOCs, sensory characteristic analyses, molecular sensory approaches, and the investigation of potential formation pathways [19,20,21,22]. However, the coordinated evolution of flavor precursors, volatile profiles, odor activity, and sensory characteristics across successive roasting stages remains insufficiently characterized. In particular, understanding how changes in soluble sugars and chlorogenic acids correspond with the development of aroma-active compounds and sensory transitions may provide deeper insights into the evolution of coffee flavor. Although geographical origin and cultivar can influence the development of coffee flavor, the roasting-induced flavor evolution of Arabica (Catimor variety) coffee from Baoshan remains to be further characterized [31]. Therefore, a stage-resolved integrated evaluation—combining analyses of soluble sugars, chlorogenic acids, VOCs, and odor activity values (OAVs); chemometric analysis; and sensory attribute assessments—is needed to characterize the dynamic development of coffee flavor during roasting. We hypothesized that progressive roasting induces coordinated changes in flavor precursors, aroma-active compounds, and sensory characteristics, and that integrating these complementary measurements can reveal major transitions in coffee flavor development that cannot be fully captured by any single analytical approach.

In this study, Arabica (Catimor variety) green coffee beans from Baoshan, Yunnan Province, China, were used as the raw material. The unroasted green beans were designated as S0, and eight successive roasting stages (S1–S8) were established to capture the progression of roasting from the late phase of first crack to the onset of visible surface oil. HPLC, HS-SPME-GC–MS, and sensory evaluation were employed to track changes in the chemical composition and sensory quality of coffee throughout the roasting process. The relationships among flavor precursors, aroma-active compounds, and sensory attributes were further explored to identify potential indicators associated with major transitions in coffee flavor development. This study is expected to provide a scientific basis for improving roasting control and quality management and offer practical insights across the coffee value chain.

## 2. Materials and Methods

### 2.1. Chemicals and Materials

Unless otherwise specified, all chemicals used in the experiments were of analytical grade. The chemical standards used in the experiments were purchased from Sigma-Aldrich (Saint-Quentin-Fallavier, France).

The coffee beans used in this study were produced in Lujiang Town, Baoshan City, Yunnan Province, China. Coffee roasting was performed using a Sandouke X3 coffee roaster (Zhuzhou, China). The unroasted green coffee beans were designated as S0. For the roasted samples, green coffee beans were divided into separate portions and independently roasted to eight target roasting degrees. The resulting samples were designated as S1–S8 according to increasing roasting degree, from lightest to darkest, based on the roasting characteristics and measured bean color values. Three independent roasting runs were performed for each roasting stage. Detailed sample characteristics for S1–S8 are provided in Table S1. After roasting, the coffee beans were rapidly cooled to room temperature and stored in sealed coffee bags until further analysis.

### 2.2. Analysis of Soluble Sugars

Soluble sugars were identified and quantified by HPLC according to our previously reported method with minor modifications [32]. Soluble sugars were separated on a Phenomenex Luna Omega SUGAR column (4.6 × 250 mm, 3 μm) (Phenomenex, Torrance, CA, USA), with water (A) and acetonitrile (B) used as the mobile phases. The proportion of B was decreased from 93% to 75% during 0–1 min and maintained at 75% until 15 min. It was then reduced to 60% at 20 min and 40% at 21 min, before returning to 93% at 22 min; this composition was maintained until 30 min for column re-equilibration. The separation was carried out at 30 °C with a flow rate of 1.0 mL/min and an injection volume of 10 μL. An evaporative light-scattering detector was operated at 350 kPa nebulizer pressure and 40 °C. Compounds were identified by comparing retention times with authentic standards analyzed under identical chromatographic conditions, while quantification was carried out using external calibration curves (Table S2). The limit of detection (LOD) and limit of quantification (LOQ) are 1000 and 2500 mg/kg, respectively. The results are expressed on a dry-weight basis.

### 2.3. Analysis of Chlorogenic Acids

Chlorogenic acids were identified and quantified by Ultra-performance liquid chromatography (UPLC) according to the Chinese national standard GB/T 43733-2024 with minor modifications [33]. Chromatographic separation was performed using a Waters ACQUITY UPLC HSS T3 column (2.1 mm × 100 mm, 1.8 μm) (Waters, Milford, MA, USA). Chromatographic separation was achieved using a binary mobile phase composed of 0.1% (*v*/*v*) formic acid in water (A) and 80% (*v*/*v*) acetonitrile in water (B). The proportion of solvent A was programmed as follows: 89% (0–10 min), 84% (10–18 min), 84–75% (18–22 min), 75–74% (22–30 min), 74–54% (30–40 min), and 54–40% (40–42 min), followed by returning to 89% A from 42 to 50 min for column re-equilibration. The analysis was conducted at 30 °C with a flow rate of 0.2 mL/min and an injection volume of 1 μL, and UV detection was performed at 330 nm. Compounds were identified by comparing retention times with authentic standards analyzed under identical chromatographic conditions, while quantification was carried out using external calibration curves (Table S2). The LOD and LOQ are 0.6 and 1.5 mg/kg, respectively. The results are expressed on a dry-weight basis.

### 2.4. Analysis of Caffeine

Caffeine was identified and quantified using UPLC, according to the Chinese national standard GB 5009.139-2014 with minor modifications [34]. Chromatographic separation was performed using an Agilent ZORBAX SB C18 column (150 mm × 4.6 mm, 5 μm). The mobile phase consisted of methanol and water at a volume ratio of 24:76 (*v*/*v*). The analysis was conducted at 25 °C with a flow rate of 1 mL/min and an injection volume of 10 μL, and UV detection was performed at 272 nm. Caffeine was identified by comparing retention times with authentic standard analyzed under identical chromatographic conditions, while quantification was carried out using an external calibration curve (Table S2). The LOD and LOQ are 18 and 54 mg/kg, respectively. The results are expressed on a dry-weight basis.

### 2.5. Analysis of VOCs

The extraction and determination of VOCs were performed based on previous methods [19,35]. A CTC CombiPAL autosampler (CTC Analytics, Zwingen, Switzerland) fitted with a 2 cm DVB/CAR/PDMS SPME fiber (50/30 μm; Supelco, Bellefonte, PA, USA) was used for headspace sampling. For each analysis, ground coffee powder (2.0 g) was transferred into a 20 mL headspace vial, followed by the addition of NaCl (2.0 g), deionized water (1 mL), and 10 μL of internal standard solution (4-methyl-2-pentanol, 1.02 g/L). Before its first use, the SPME fiber was conditioned at 270 °C for 30 min and subsequently conditioned for 10 min before each extraction. The vial was equilibrated at 50 °C for 30 min, and the SPME fiber was then inserted into the headspace for extraction at 50 °C for an additional 30 min.

VOC separation and identification were conducted using an Agilent 8890 GC system coupled with a 5977B mass spectrometer (Agilent Technologies, Santa Clara, CA, USA). Chromatographic separation was achieved on an HP-INNOWAX capillary column (60 m × 0.25 mm, 0.25 μm; Santa Clara, CA, USA). Prior to analysis, the SPME fiber was thermally desorbed in the GC inlet at 250 °C for 8 min under splitless conditions. Helium (>99.999%) was employed as the carrier gas at a constant flow rate of 1.0 mL/min, and the inlet temperature was set at 250 °C. The oven program started at 44 °C, followed by an increase to 107 °C at 3 °C/min with a 1 min hold. The temperature was subsequently raised to 155 °C at the same rate and finally increased to 230 °C at 20 °C/min, where it was maintained for 15 min. Mass detection was performed under electron ionization conditions at 70 eV. The ion source, quadrupole, and transfer line temperatures were maintained at 230, 150, and 230 °C, respectively. Full-scan acquisition was carried out over an *m*/*z* range of 30–350.

Data processing was performed using MassHunter Workstation software (version 12.0; Agilent Technologies, Santa Clara, CA, USA). The identification of VOCs was based on mass spectral matching against the NIST20 library and comparison of experimental retention indices (RIs) with reported values in the literature. Retention indices were calculated using a homologous series of n-alkanes analyzed under the same chromatographic conditions. VOCs with a NIST similarity score ≥ 80% and an RI deviation within ±10 RI units were identified based on the agreement of mass spectral and RI information. The identities of compounds with available authentic standards were further confirmed by comparing their retention times and mass spectra with those of the corresponding standards. For compounds without authentic standards, identification was established based on the combined evidence of mass spectral similarity and RI agreement. VOCs with available authentic standards were quantified using calibration curves established from the analyte-to-internal-standard response ratios under the same analytical conditions (Table S3). For compounds without authentic standards, semi-quantification was conducted using the response ratio relative to the internal standard.

### 2.6. Calculation of OAVs

The sensory relevance of each VOC was estimated from its OAV, calculated as the ratio of its concentration to the corresponding odor threshold [36]:

OAV = C/T(1)

where C represents the VOC concentration (mg/kg), and T is its odor threshold (mg/kg). Odor thresholds used for OAV calculation were obtained mainly from the literature and a reference book [5,37], while odor descriptors were compiled from the literature [5] and the Flavornet database (https://www.flavornet.org/flavornet.html, accessed on 20 August 2026).

### 2.7. Sensory Evaluation

Sensory evaluation was conducted according to the traditional Specialty Coffee Association (SCA) cupping protocol, with additional reference to the local document “Quality Requirements for Geographical Indication Products—Baoshan Arabica Coffee” issued in Yunnan Province, China. Coffee beans from each roasting stage were ground separately immediately before sensory evaluation. To minimize carryover between samples, a small portion of the corresponding coffee sample was passed through the grinder and discarded before grinding the sample used for cupping. The coffee beans were ground to pass through a No. 20 sieve (0.85 mm aperture). Five independent cupping bowls were prepared for each sample using a coffee-to-water ratio of 1:18 (*w*/*w*), and cupping preparation was completed within 30 min after grinding. Before brewing, the dry fragrance of the ground coffee was evaluated, and the predominant aroma descriptors were recorded. Hot water at a temperature above 90 °C was then added to each cupping bowl, and the aroma released after water addition was evaluated. After 4 min of steeping, the crust was broken, and the aroma was reassessed immediately. As the coffee infusion gradually cooled, the samples were slurped from stainless-steel cupping spoons and evaluated at three temperature ranges: high (>50 °C), medium (35–50 °C), and low (20–35 °C). Representative aroma and flavor descriptors were recorded throughout the sensory evaluation.

The sensory attributes evaluated included fragrance/aroma, flavor, aftertaste, acidity, body, uniformity, balance, clean cup, sweetness, overall, and final score. Fragrance/aroma, flavor, aftertaste, acidity, body, balance, sweetness, and overall were scored on a 6–10-point scale, with increments of 0.25 points. Uniformity and clean cup were evaluated based on the consistency of sensory performance among the five replicate cups. For each sample, individual sensory attribute scores were summed after deduction of any defect scores, and the mean score across all panelists was reported as the final score. Sensory evaluation was conducted at the Baoshan Institute for Inspection and Testing, Baoshan, Yunnan Province, China, by five trained adult sensory panelists. According to the institutional regulations, ethical approval was not required for this routine food sensory evaluation. Before the sensory test, panelists were briefed on the study objectives and evaluation procedures.

### 2.8. Data Analysis

For each sample, all analytical measurements were conducted in triplicate, and the mean value was calculated and used as the representative value for each independent roasting batch. Statistical analyses were performed based on the three independent roasting batches. Data were organized and processed in Excel 2016 (Microsoft Corp., Redmond, WA, USA), and statistical analyses were carried out with IBM SPSS Statistics 27.0 (IBM Corp., Armonk, NY, USA). Differences were considered significant at *p* ≤ 0.05. Data visualization was conducted using MetaboAnalyst 6.0 (https://www.metaboanalyst.ca, accessed on 30 August 2026) and Origin 2024 (OriginLab Corp., Northampton, MA, USA).

## 3. Results and Discussions

### 3.1. Soluble Sugar, Chlorogenic Acid, and Caffeine Profiles of Coffee Beans at Different Roasting Stages

**Figure 1:**
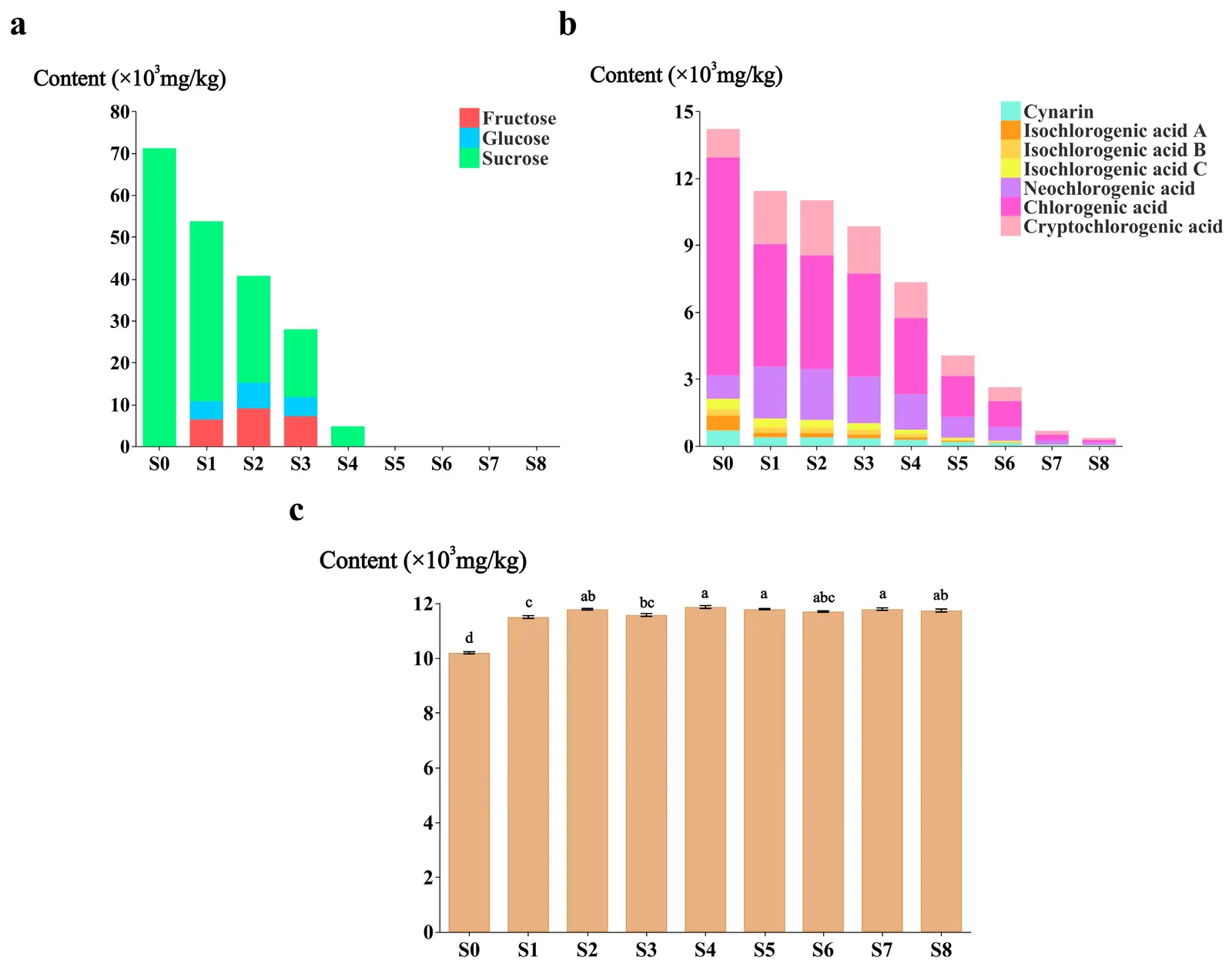
Soluble sugar, chlorogenic acid, and caffeine profiles of coffee beans at different roasting stages. (**a**) Vertical stacked bar chart showing the concentrations of individual soluble sugars across roasting stages. (**b**) Vertical stacked bar chart showing the concentrations of individual chlorogenic acids across roasting stages. (**c**) Bar chart showing the concentration of caffeine across roasting stages. Different lowercase letters indicate significant differences among treatments (*p* < 0.05).

Soluble sugars are important aroma precursors during coffee roasting, and their transformation is closely associated with caramelization and Maillard reactions [19]. As shown in Figure 1a, three soluble sugars—namely, sucrose, fructose, and glucose—were detected in the coffee samples across the different roasting stages. Overall, the total soluble sugar content progressively decreased with increasing roasting degree. The highest level was observed at S0, in which sucrose was the predominant soluble sugar. Once roasting commenced, sucrose decreased rapidly and became undetectable at S5. This result indicated its extensive consumption and degradation during progressive thermal processing. In contrast, neither fructose nor glucose was detected at S0. Both of these reducing sugars became detectable after the onset of roasting and reached their highest levels at S2, followed by a continuous decline until they were no longer detectable at S4. Their transient accumulation during the early roasting stages may be associated with sucrose transformation under thermal conditions, including possible hydrolysis, which could temporarily increase the availability of fructose and glucose [24]. Alternatively, the observed changes in sugar concentrations may reflect the combined effects of sucrose transformation, thermal degradation, and their involvement in subsequent roasting reactions [38]. As roasting progressed, the decline in reducing sugar levels was consistent with their consumption or transformation during thermal reactions, including Maillard and caramelization reactions [39]. This pattern is consistent with previous studies describing the progressive transformation and depletion of soluble sugars during coffee roasting [13,20]. Notably, fructose and glucose were no longer detectable at S4, and this was followed by the disappearance of sucrose at S5. This compositional shift may be closely associated with subsequent changes in aroma development during roasting.

Chlorogenic acids contribute directly to coffee taste and undergo isomerization, degradation, and other thermal transformations during roasting, thereby influencing flavor development [40,41]. A total of seven chlorogenic acids were detected across the different roasting stages of coffee samples, including cynarin; isochlorogenic acids A, B, and C; neochlorogenic acid; chlorogenic acid; and cryptochlorogenic acid (Figure 1b). Overall, the total chlorogenic acid content decreased markedly as roasting progressed, reflecting the susceptibility of chlorogenic acids to progressive thermal treatment. Chlorogenic acid was the predominant compound at S0, accounting for 68.72% of the total chlorogenic acids. Its content decreased continuously with increasing roasting degree and reached the lowest level at S8, corresponding to a 98.54% reduction relative to S0. Nevertheless, chlorogenic acid still accounted for 37.42% of the total chlorogenic acids at S8, indicating that it remained one of the major chlorogenic acids even after extensive roasting. A generally similar decreasing trend was observed for cynarin and isochlorogenic acids A, B, and C; in particular, the three isochlorogenic acids declined to very low levels after S7, indicating substantial depletion under severe roasting conditions. In contrast, neochlorogenic acid and cryptochlorogenic acid did not decrease immediately after roasting commenced. Their contents initially increased and reached the maximum levels at S2, followed by a progressive decline to their lowest levels at S8. This transient accumulation indicates that the evolution of chlorogenic acids during roasting may involve processes beyond direct thermal degradation, including possible isomerization or interconversion among chlorogenic acid isomers and differences in their thermal stability [42]. Under high-temperature conditions, chlorogenic acids can undergo further thermal transformation and contribute to the formation of volatile phenolic compounds [40]. These compounds are important contributors to coffee’s smoky and roasted notes, but their impact on coffee aroma can be either favorable or undesirable depending on their levels. At moderate levels, their formation may enrich roasted aroma complexity, whereas excessive accumulation may contribute to overly smoky and burnt–bitter sensory characteristics [43]. However, the dynamic evolution of coffee aroma quality during roasting requires further verification in combination with the analysis of VOCs.

As shown in Figure 1c, the caffeine content was markedly higher in the roasted coffee beans than in the green coffee beans. All roasted samples exhibited significantly higher caffeine levels than the S0 samples (*p* < 0.05). Among the roasted samples, caffeine content varied within a relatively narrow range and showed no clear increasing or decreasing trend with increasing roasting degree. The highest value was observed at S4, although it did not differ significantly from the value obtained at S2, S5, S6, S7, or S8 (*p* > 0.05). Previous studies have similarly shown that caffeine remains relatively stable during roasting, although slight variations among roasting degrees have been reported [20,44,45]. Although S4 showed the highest mean caffeine content, it did not differ significantly from several other roasted samples. Thus, caffeine content alone was insufficient to identify a roasting stage with uniquely greater stimulant potential [46,47,48].

### 3.2. VOC Profiles of Coffee Beans at Different Roasting Stages

**Figure 2:**
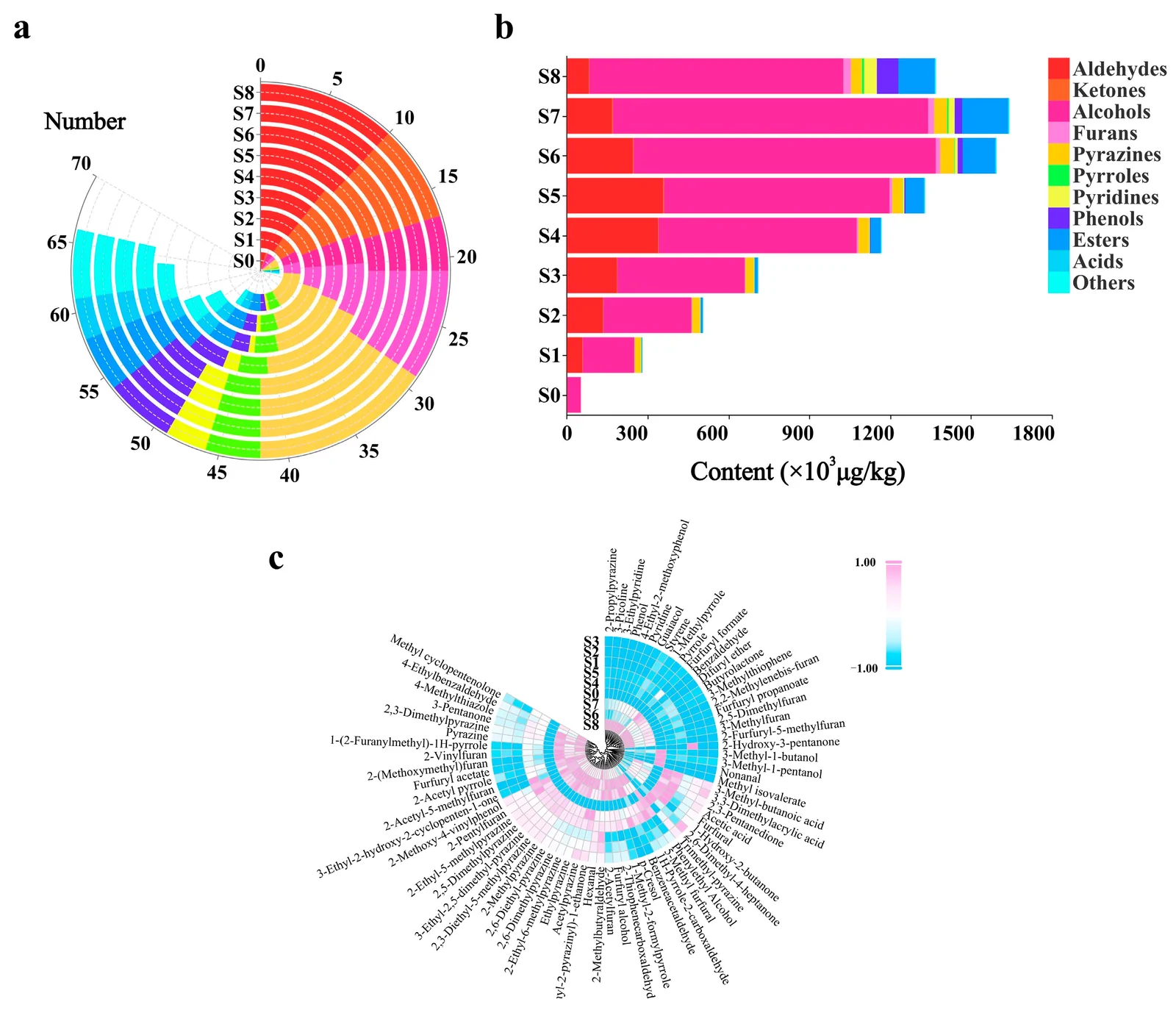
VOC profiles of coffee beans at different roasting stages. (**a**) Radial stacked bar chart showing the numbers of detected VOCs in different chemical classes across roasting stages. (**b**) Horizontal stacked bar chart showing the concentrations of different VOC classes across roasting stages. (**c**) Circular clustered heatmap showing the normalized concentration patterns of individual VOCs across roasting stages.

Detailed qualitative results on the VOCs identified in this study are provided in Table S4. A total of 70 VOCs were identified across all coffee samples, including 11 aldehydes, 8 ketones, 4 alcohols, 8 furans, 14 pyrazines, 4 pyrroles, 3 pyridines, 5 phenols, 5 esters, 3 acids, and 5 other compounds (Figure 2a). Relatively few VOCs were detected at S0, with pyrazines representing the largest group, followed by aldehydes, alcohols, and furans. As roasting progressed, the number of detected VOCs continuously increased and reached its maximum at S5, after which no further increase was observed. At S5, pyrazines remained the most diverse class, followed by aldehydes, ketones, and furans. Based on the qualitative results, the VOCs were further quantified (Table S5). The total VOC concentration rose progressively with roasting and peaked at S7 before decreasing at S8 (Figure 2b). The different peak stages of VOC number and total concentration indicate that volatile evolution during roasting involves changes in both VOC diversity and overall abundance. Therefore, the stage with the highest VOC diversity does not necessarily correspond to the stage with the highest total volatile concentration. At S0, alcohols comprised the most abundant volatile class, followed by aldehydes and esters. At S7, alcohols remained the predominant class, followed by esters and aldehydes. This divergence suggests that the early-to-middle roasting stages are mainly characterized by the formation of new VOCs and an increase in VOC diversity. After S5, further roasting primarily promoted the accumulation and redistribution of previously formed VOCs, together with the transformation and degradation of some compounds. Interestingly, different classes of VOCs exhibited distinct accumulation patterns throughout roasting. Aldehydes reached their maximum concentration at S5 and subsequently declined markedly, whereas pyrazines peaked at S6, and alcohols and esters reached their highest levels at S7. In contrast, furans, pyrroles, pyridines, and phenols continued to accumulate during the later roasting stages and reached their maximum concentrations at S8. The relative abundance of different VOC classes further revealed a dynamic shift in volatile composition during roasting (Figure S1). Early roasting stages showed a higher proportion of alcohols, aldehydes, and esters, whereas advanced roasting resulted in an increased relative contribution of furans, pyridines, and phenols. These results indicate that roasting not only increases the overall abundance of VOCs, but also reshapes the volatile composition. These distinct trends highlight the differential evolution of individual VOC classes during progressive thermal treatment. Therefore, further analysis of individual VOCs is necessary to better understand the stage-dependent evolution of the coffee volatile profile during roasting.

A clustered heatmap was generated based on the normalized concentrations of individual VOCs (Figure 2c). Several compounds detected at S0 decreased rapidly once roasting commenced. Methyl isovalerate, characterized by fruity and herbal odors, and nonanal, associated with fatty, citrus-like, and green notes, were no longer detected after S0. The concentrations of 3-methyl-1-butanol, described as whiskey-like, malty, and burnt, and 3-methyl-1-pentanol, characterized by fruity and wine-like notes, also decreased markedly during the early roasting stages. These changes suggest that some VOCs initially present in green coffee beans may be reduced during early roasting through volatilization, thermal degradation, or other transformation processes [6]. In contrast, another group of VOCs accumulated rapidly during the early-to-middle roasting stages. Furfural and 5-methylfurfural both reached their maximum concentrations at S5 and are commonly associated with sweet, caramel-like, bready, and coffee-like aroma. Similarly, 2,3-pentanedione and 1-hydroxy-2-butanone, characterized by buttery, creamy, nutty, and sweet notes, accumulated markedly during the middle roasting stages. Acetic acid and 3-methylbutanoic acid, associated with sour and pungent odors, also reached relatively high concentrations during these stages. Together with the pronounced depletion of soluble sugars observed during these stages, these changes are consistent with intensified sugar degradation, Maillard reactions, and related thermal reactions [24]. The volatile composition therefore gradually shifted from compounds associated predominantly with fruity and green notes toward those characterized by sweeter, caramel-like, and nutty odors. As roasting progressed into the middle-to-late stages, compounds typically associated with roasted coffee became increasingly abundant. Furfuryl alcohol, furfuryl acetate, and 2-acetylfuran showed marked accumulation and are commonly described as having caramel-like, sweet, nutty, and cocoa-like odors. Several alkylpyrazines, including 2-methylpyrazine, 2,3-dimethylpyrazine, ethylpyrazine, 2,5-dimethylpyrazine, 2,6-dimethylpyrazine, and 2-ethyl-5-methylpyrazine, reached relatively high concentrations at S6–S7 and are generally associated with nutty, coffee-like, cocoa-like, and roasted notes. By comparison, several pyridines and phenolic compounds showed stronger accumulation during advanced roasting. Pyridine and 3-ethylpyridine increased markedly during the later stages and are associated with smoky, burnt, tobacco-like, and leather-like odors. Guaiacol, phenol, and 4-ethyl-2-methoxyphenol are characterized mainly by phenolic, woody, smoky, and spicy notes, while 1-methylpyrrole and pyrrole are associated with a roasted, woody, nutty, and sweet aroma. The pronounced accumulation of these compounds at S8 indicates a substantial shift in the volatile composition toward compounds with stronger roasted, smoky, woody, and spicy odor characteristics during advanced roasting. These changes may reflect the combined effects of prolonged thermal exposure, degradation of precursor compounds, and the formation of aroma-active compounds under higher roasting intensity.

### 3.3. Odor-Active Compound Profiles of Coffee Beans at Different Roasting Stages

**Figure 3:**
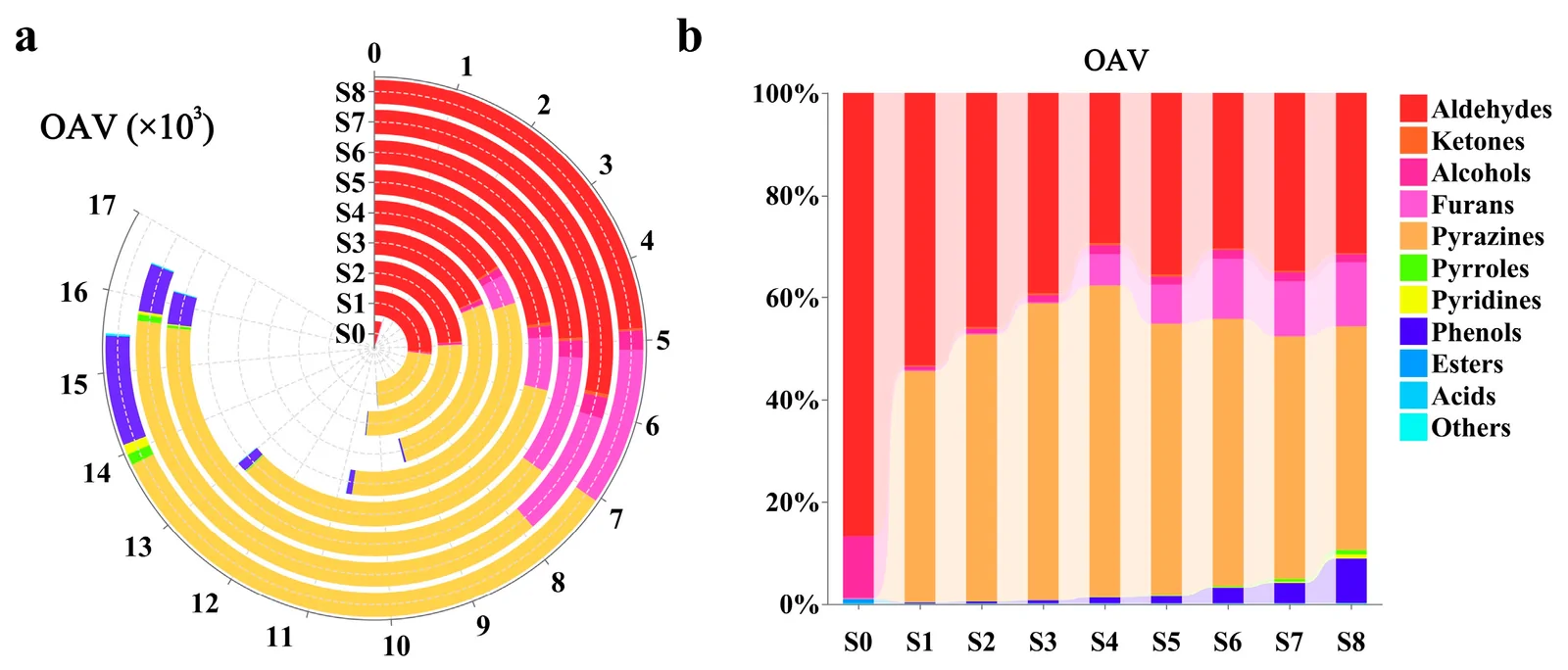
Odor activity profiles of coffee beans at different roasting stages. (**a**) Radial stacked bar chart showing the total OAV across roasting stages. (**b**) Vertical 100% stacked bar chart showing the relative contributions of different VOC classes to the total OAV across roasting stages.

To further evaluate the potential contributions of individual VOCs to coffee aroma, OAVs were calculated for all identified compounds (Table S6). Compounds with OAVs > 1 are generally considered potential contributors to the overall aroma [49]. Across all roasting stages, 45 compounds exhibited OAVs > 1 and were therefore classified as odor-active compounds. As shown in Figure 3a, the summed OAV increased markedly with roasting progression, reaching its maximum at S7 before declining slightly at S8. The relative contributions of different chemical classes to the summed OAV also changed substantially during roasting (Figure 3b). Aldehydes dominated the odor activity of green coffee beans at S0, whereas pyrazines rapidly became the predominant odor-active class after roasting began and reached their highest relative contribution at S4. With further roasting, the contributions of furans and phenols gradually increased, both becoming most prominent at S8. Similar changes have been reported in previous studies, in which pyrazines were important odor-active compounds in roasted coffee, while furans and phenols became more prominent with increasing roasting degree [50,51,52]. Overall, the OAV profile showed a clear stage-dependent evolution during roasting, characterized by changes in both overall odor activity and the relative contributions of individual VOC classes.

**Figure 4:**
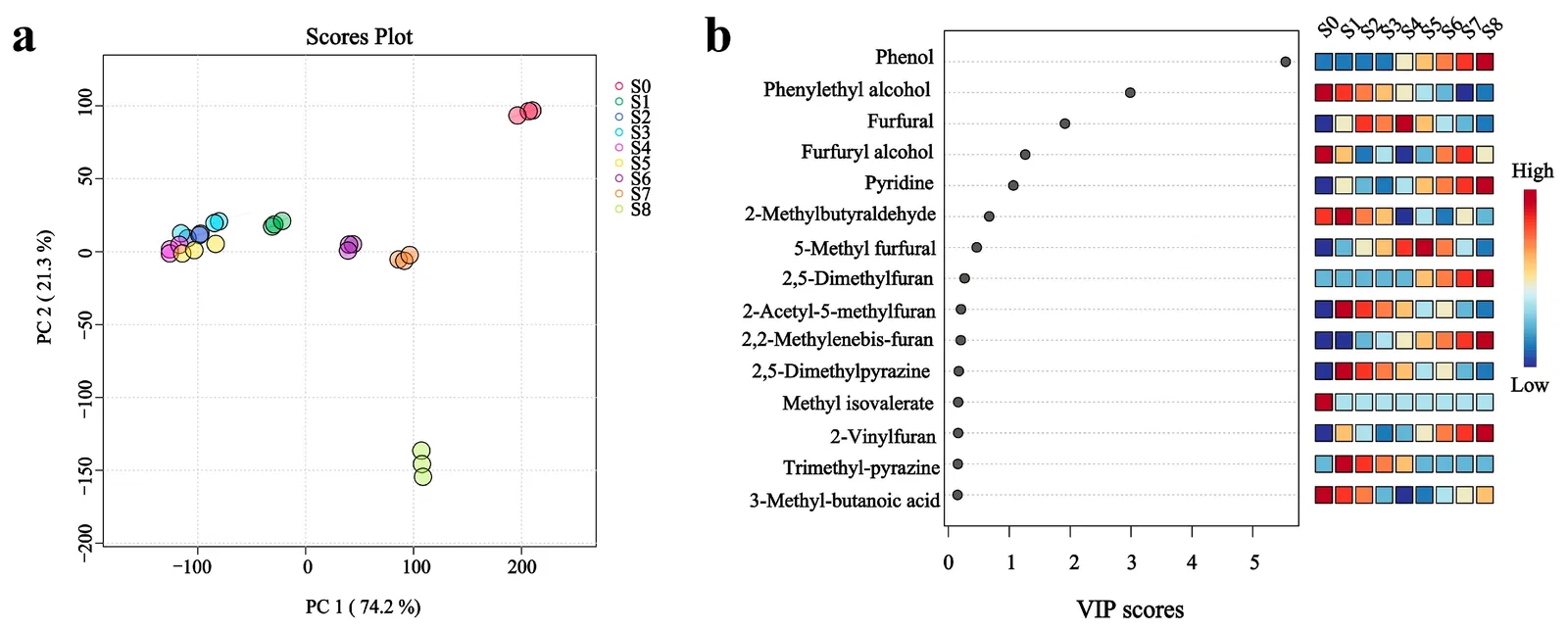
Multivariate analysis of odor-active compounds in coffee beans at different roasting stages. (**a**) PCA score plot based on 47 odor-active compounds across roasting stages. (**b**) Importance of individual odor-active compounds based on VIP scores derived from PLS-DA. The colored boxes on the right indicate their relative associations with the different roasting stages.

To further characterize differences in the aroma profiles across roasting stages, principal component analysis (PCA) was performed using these 45 odor-active compounds (Figure 4a). Together, the first two principal components accounted for 95.5% of the total variance and captured most of the variation among the samples. The S0 samples were clearly separated from all other roasted samples, reflecting the pronounced transformation of the aroma profile after roasting. Among the roasted samples, samples at S1–S4 were located relatively close to one another, whereas S5 samples began to deviate from this cluster. The positions of S5, S6, and S7 samples shifted progressively across the PCA space, while S8 samples were distinctly separated from the other roasted samples. These progressive changes indicate that the aroma profile undergoes continuous reorganization throughout roasting rather than an abrupt transition at a single stage [5]. Notably, S5 was the first stage to separate clearly from the earlier roasting stages, highlighting the S4–S5 transition as a key turning point in coffee aroma development.

Partial least squares discriminant analysis (PLS-DA) was used to identify compounds contributing to the separation among roasting stages. The relative contributions of the 45 odor-active compounds were assessed using variable importance in projection (VIP) scores. The compounds with VIP > 1 were considered potential discriminant compounds. As shown in Figure 4b, phenol, phenylethyl alcohol, furfural, furfuryl alcohol, and pyridine contributed the most to the discrimination among roasting stages. These compounds exhibited distinct stage-related patterns. Phenylethyl alcohol, characterized by honey-like, spicy, rose-like, and lilac-like notes, was primarily associated with green coffee beans and the early roasting stages (S0–S2). A similar enrichment of phenylethyl alcohol in very lightly roasted coffee has been reported previously [20]. Furfural, which is commonly associated with sweet, bready, and caramel-like notes, showed a strong association with the intermediate roasting stages (S3–S5). Furfural can be generated through sugar degradation and Maillard reaction-related pathways, while S3–S5 coincided with substantial transformation and depletion of soluble sugars (Figure 1a) [53]. Furfuryl alcohol, characterized by burnt, sweet, bready, and caramel-like notes, has previously been identified as an important odor-active compound in Yunnan Arabica coffee [20] and was mainly associated with the middle-to-late roasting stages in this study. In contrast, phenol and pyridine were predominantly associated with the later roasting stages (S6–S8). Phenol is typically characterized by phenolic and medicinal-like odors, whereas pyridine is associated mainly with smoky, burnt, and coffee-like notes. These compounds may enhance the typical aroma of dark roast coffee at moderate levels, but excessive accumulation can shift the sensory profile toward stronger smoky, burnt, and harsh sensory characteristics [21]. Nevertheless, the sensory consequences of these compositional changes require further evaluation in combination with cupping results.

### 3.4. Sensory Profiles of Coffee Beans at Different Roasting Stages

As shown in Table 1, the final cupping score exhibited a clear increase followed by a decline as roasting progressed. Samples at S0 and S1 received relatively low scores, after which the cupping score gradually increased with increasing roasting degree and reached its maximum at S4. With further roasting, the score progressively declined and reached its lowest value at S8. These results indicate that sensory quality is strongly dependent on roasting degree. Moderate roasting was associated with improved flavor development and overall sensory balance, whereas excessive roasting resulted in a progressive deterioration in cup quality [9].

Regarding the specific sensory characteristics, samples at S0–S1 were characterized by relatively limited development of typical roasted coffee flavors. Samples at S0 were dominated by grassy, raw soybean-like, and citrus-like notes, whereas samples at S1 exhibited white floral, red wine-like, and raw pea-like characteristics. These descriptors indicate that the characteristic sweet, nutty, and roasted notes of roasted coffee had not yet been fully developed at these early stages. A marked sensory transition became evident from S2 onward. Samples at S2 were characterized primarily by strawberry-, longan-, and candy-like notes, accompanied by increasingly pronounced fruity and sweet characteristics. At S3, the sensory profile shifted toward roasted peanut-, sugar-, cereal-, and caramel-like notes, indicating the progressive development of more typical roasted coffee characteristics. Samples at S4 showed prominent nutty, roasted hazelnut-like, and caramel-like notes, with a persistent aftertaste and noticeable lingering sweetness. Consistent with these sensory characteristics, samples at S4 also received relatively high scores for fragrance/aroma, flavor, body, balance, and overall impression, suggesting a more harmonious and well-integrated sensory profile.

Beyond S4, the sensory profile gradually shifted from balanced flavor development toward quality deterioration. Samples at S5 retained the relatively pleasant dark chocolate and spicy notes, although a more pronounced pine-like roasted characteristic also began to emerge. By S6, smoky, burnt, and bitter sensations became increasingly prominent, suggesting that the intense sensory characteristics generated during deeper roasting had begun to disrupt the overall flavor balance. Samples at S7 were dominated by smoky, charcoal-like, and pine-like notes, accompanied by a pronounced bitter aftertaste. At S8, charcoal-like, medicinal-bitter, and over-roasted characteristics became even more evident, resulting in a marked loss of flavor complexity, balance, and overall sensory quality.

Under the other roasting conditions used in this study, samples at S4 exhibited the most favorable overall sensory quality, while the transition from S4 to S5 represented a critical interval at which flavor development began to shift toward sensory deterioration. This pattern was consistent with the changes observed in the odor-active compound profiles (Figure 3a). Therefore, the composition and relative contributions of key odor-active compounds played an important role in shaping the stage-dependent sensory characteristics of roasted coffee.

## 4. Conclusions

This study systematically characterized the dynamic changes in non-volatile constituents, odor-active compounds, and cup quality of Arabica coffee from green beans through eight successive roasting stages. The total contents of soluble sugars and chlorogenic acids progressively decreased with increasing roasting degree. In contrast, VOC diversity increased until S5, while the total VOC concentration continued to rise and reached its maximum at S7. The OAV analysis identified 47 potential odor-active compounds with OAVs > 1, among which phenylethyl alcohol, furfuryl alcohol, furfural, phenol, and pyridine were identified as the major discriminant makers across roasting stages. Sensory evaluation further showed that cup quality progressively improved from the early roasting stages and reached its optimum at S4, where nutty, roasted hazelnut-like, and caramel-like notes were well balanced. With further roasting, smoky and burnt–bitter characteristics became increasingly pronounced, resulting in a decline in cup quality. Therefore, the transition from S4 to S5 can be regarded as a critical turning point in the evolution of coffee aroma during roasting. These findings provide useful insights into aroma development during continuous roasting and offer a basis for quality control of roasted coffee.

## Figures and Tables

**Table 1:** Sensory attributes and cupping scores of coffee beans at different roasting stages.

Sample	S0	S1	S2	S3	S4	S5	S6	S7	S8
Fragrance/Aroma	7.25	7.50	7.75	7.50	7.75	7.25	7.25	7.00	6.50
Aroma quality	Underdeveloped	Fermented	Berry	Nutty	Nutty	Cheesy	Cheesy	Smoky	Charred
Flavor	7.25	7.25	7.50	7.50	7.50	7.25	7.00	6.75	6.00
Aftertaste	7.25	7.50	7.25	7.25	7.50	7.25	7.25	6.50	6.50
Acidity	7.50	7.25	7.50	7.75	7.50	7.25	7.25	6.50	6.00
Body	7.25	7.25	7.25	7.50	7.50	7.50	7.25	7.25	6.75
Uniformity	10.00	10.00	10.00	10.00	10.00	10.00	10.00	10.00	10.00
Balance	7.25	7.25	7.50	7.50	7.50	7.25	7.25	6.75	6.25
Clean cup	10.00	10.00	10.00	10.00	10.00	10.00	10.00	10.00	10.00
Sweetness	10.00	10.00	10.00	10.00	10.00	10.00	10.00	10.00	10.00
Overall	7.25	7.25	7.50	7.50	7.50	7.25	7.25	6.75	6.25
Final score	81.00	81.25	82.25	82.50	82.75	81.00	80.50	77.50	74.25
Sensory descriptors	Grassy, raw soybean, citrus	Dry aroma with white floral and red wine notes, green pea-like note (underdeveloped), slight sweet aftertaste	Strawberry, longan, candy-like, slightly fermented, short aftertaste	Roasted peanut, sucrose-like, cereal, caramel; slightly dry and astringent finish	Nutty, roasted hazelnut, caramel; long aftertaste with sweetness	Dry aroma, dark chocolate, spice, pine wood	Smoky, dark chocolate, pine wood, burnt bitterness	Smoky, charcoal, pine wood, bitter aftertaste	Medicinal bitterness, over-roasted, lacking flavor

## Data Availability

Data will be made available upon request.

## Supplementary Materials

The following supporting information can be downloaded at: https://www.mdpi.com/article/10.3390/foods15193569/s1, Figure S1: Dynamic changes in volatile composition during coffee roasting; Table S1: Characteristics of coffee beans at different roasting stages; Table S2: Calibration curves for quantification of soluble sugars and chlorogenic acids for coffee beans at different roasting stages; Table S3: Calibration curves for quantification of VOCs for coffee beans at different roasting stages; Table S4: Identification results and odour descriptions of VOCs for coffee beans at different roasting stages; Table S5: Quantitative results of VOCs for coffee beans at different roasting stages; Table S6: OAVs of VOCs for coffee beans at different roasting stages.

## Abbreviations

The following abbreviations are used in this manuscript:

HPLC	High-performance liquid chromatography
UPLC	Ultra-performance liquid chromatography
LOD	Limit of detection
LOQ	Limit of quantification
HS-SPME-GC–MS	Headspace solid-phase microextraction and Gas chromatography–mass spectrometry
VOC	Volatile compound
OAV	Odor-active value
PCA	Principal component analysis
PLS-DA	Partial least squares discriminant analysis
