# Supplementary material for: From Chemical Transformations to Cup Quality: Integrated Insights into Key Non-Volatile Compounds, Aroma-Active Compounds and Sensory Evolution During Continuous Roasting Stages in Yunnan Arabica Coffee
Source: Foods. 2026 Oct 8;15(19):3569. doi: 10.3390/foods15193569 (PMC13665445; doi:10.3390/foods15193569)
Supplement: Supplementary file 1 [file foods-15-03569-s001.zip › foods-4596957-supplementary.pdf]

From Chemical Transformations to Cup Quality: Integrated Insights into Key Non-Volatile Compounds, Aroma-Active Compounds and Sensory Evolution During Continuous Roasting Stages in Yunnan Arabica Coffee

Yizhi Chen [1,2], Siqian Guo [1,2], Yilin Wang [1], Xianwen Wang [3,4], Tao Tong [1,3,4,5], Wentao Gao [1,2], Kexi Ma [1] and Liyan Ma [1,3,4,5,*]

[1] College of Food Science & Nutritional Engineering, China Agricultural University, No. 17 Tsinghua Dong Road, Beijing 100083, China; chenyizhi10200820@163.com (Y.C.); guosiqian2022@163.com (S.G.); 15887170929@163.com (Y.W.); tongtao1028@cau.edu.cn (T.T.); gaowentao636@163.com (W.G.); makexi2009@163.com (K.M.)

[2] CAU Sichuan Chengdu Advanced Agricultural Industrial Institute, No. 515 Xingyuan 8th Road, Chengdu 611430, China

[3] Baoshan Inspection and Testing Institute, Baoshan 678000, China; awenchina@163.com

[4] Key Laboratory of Coffee Quality and Safety, State Administration for Market Regulation, Baoshan 678000, China

[5] Inspection and Testing Center for Agricultural Products Quality, Ministry of Agriculture and Rural Affairs, Beijing 100083, China

* Correspondence: lyma1203@cau.edu.cn

Figure S1 Dynamic changes in volatile composition during coffee roasting.

Table S1 Characteristics of coffee beans at different roasting stages.

Table S2 Calibration curves for quantification of soluble sugars and chlorogenic acids for coffee beans at different roasting stages.

Table S3 Calibration curves for quantification of VOCs for coffee beans at different roasting stages.

Table S4 Identification results and odour descriptions of VOCs for coffee beans at different roasting stages.

Table S5 Quantitative results of VOCs for coffee beans at different roasting stages.

Table S6 OAVs of VOCs for coffee beans at different roasting stages.

Figure S1 Dynamic changes in volatile composition during coffee roasting.

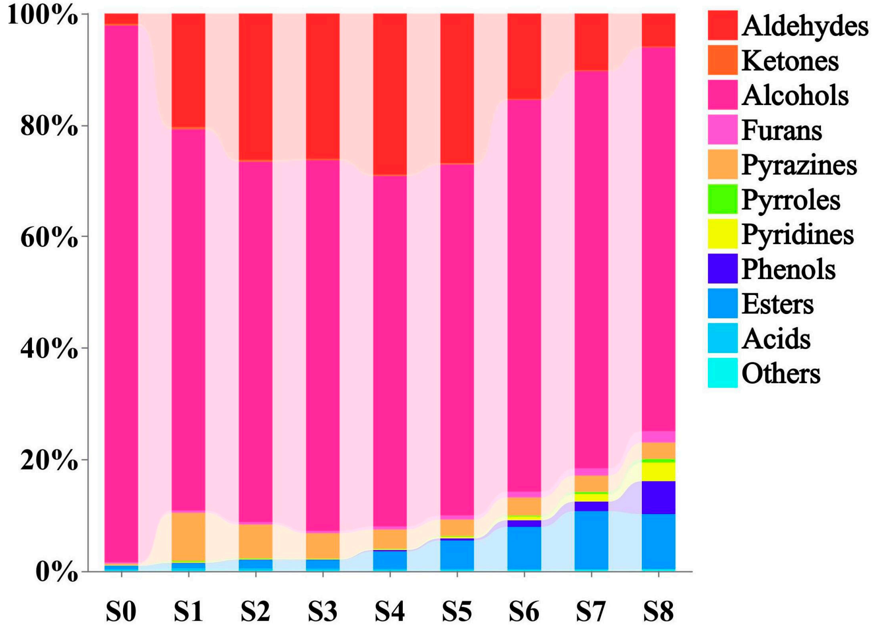

Table S1 Characteristics of coffee beans at different roasting stages.

No.	Roasting stages	Discharge criterion	Color value
1	S1	Near the end of intensive first crack	95.03±4.40
2	S2	Around the end of first crack	84.53±1.56
3	S3	After the end of first crack	75.03±0.68
4	S4	Quiescent period between first and second crack	61.90±0.79
5	S5	Beginning of second crack	48.47±0.25
6	S6	Before intensive second crack	39.90±1.73
7	S7	During intensive second crack	26.77±0.15
8	S8	Initial oil migration to the bean surface	21.80±0.00

Table S2 Calibration curves for quantification of soluble sugars and chlorogenic acids for coffee beans at different roasting stages.

No.	Categories	RT (min)	Standard curves (mg/L)	R^2	Range (mg/L)
1	Fructose	8.857	y = 10.007x - 369.12	0.9992	1000-2000
2	Glucose	9.808	y = 12.253x - 752.99	0.9997	100-2000
3	Sucrose	12.703	y = 11.268x - 609.51	0.9995	100-2000
1	Neochlorogenic acid	5.035	y=64.351x+22.06	0.9998	1-50
2	Chlorogenic acid	10.058	y=76.739x+347.89	0.9991	10-500
3	Cryptochlorogenic acid	11.373	y=74.290x-3.05	0.9985	1-50
4	Cynarin	18.932	y=85.380x+0.38	0.9999	1-50
5	Isochlorogenic acid B	27.522	y=102.680x+0.42	0.9999	1-50
6	Isochlorogenic acid A	28.779	y=113.338x+3.26	0.9999	1-50
7	Isochlorogenic acid C	31.637	y=95.400x-24.73	0.9999	1-50
1	Caffeine	6.135	y = 141.76x + 540.85	0.9994	5-200

Table S3 Calibration curves for quantification of VOCs for coffee beans at different roasting stages.

No.	Categories	RT (min)	Standard curves (mg/L)	R^2	Range (mg/L)
1	Furfural	27.28	y = 0.067x + 0.0029	0.9986	1-100
2	2,3-Diethyl-5-methylpyrazine	28.24	y = 0.237x - 0.0014	0.9986	0.1-4
3	Benzaldehyde	29.90	y = 0.1057x + 0.0429	0.9999	0.1-4
4	Furfuryl acetate	30.30	y=0.095x - 0.0123	0.9974	1-60
5	5-Methyl furfural	31.97	y = 0.1147x + 0.0241	0.9996	0.1-80
6	Furfuryl alcohol	35.34	y = 0.016x - 0.0607	0.9995	5-1000
7	Phenylethyl Alcohol	41.88	y = 0.0192x - 0.0028	0.9815	1-4
8	Phenol	43.08	y = 0.0452x + 0.0109	0.9953	5-40

Table S4 Identification results and odour descriptions of VOCs for coffee beans at different roasting stages.

Categories	No.	Name	RT (min)[1]	CAS	RI	LRI	MF	Method[2]	Odour descriptions[3]
Aldehydes	1	2-Methylbutyraldehyde	6.87	96-17-3	915	920	$C_5H_{10}O$	MS+RI	Pungent, coffee-like, cocoa-like, fruity
	2	Hexanal	11.01	66-25-1	1079	1080	$C_6H_{12}O$	MS+RI+STD	Grassy, green, fresh, fatty
	3	Nonanal	23.86	124-19-6	1397	1394	$C_9H_{18}O$	MS+RI+STD	Fatty, citrus, green
	4	Furfural	27.28	98-01-1	1474	1471	$C_5H_4O_2$	MS+RI+STD	Sweet, bready, caramel-like
	5	Benzaldehyde	29.90	100-52-7	1536	1539	C_7H_6O	MS+RI+STD	Almond-like, fruity, cherry-like, powdery, nutty
	6	5-Methyl furfural	31.97	620-02-0	1585	1586	$C_6H_6O_2$	MS+RI+STD	Sweet, caramellic, bready, brown, coffee-like
	7	1-Methyl-2-formylpyrrole	34.00	1192-58-1	1634	1632	C_6H_7NO	MS+RI	Bitter, almond, nutty
	8	Benzeneacetaldehyde	34.90	122-78-1	1657	1655	C_8H_8O	MS+RI+STD	Floral, rose, cherry-like
	9	2-Thiophenecarboxaldehyde	36.96	98-03-3	1710	1718	C_5H_4OS	MS+RI	Sulfur
	10	4-Ethylbenzaldehyde	38.60	4748-78-1	1761	1730	$C_9H_{10}O$	MS+RI+STD	Sweet
	11	1H-Pyrrole-2-carboxaldehyde	43.43	1003-29-8	2008	2010	C_5H_5NO	MS+RI	Burnt, roasted, smoky
Ketones	1	3-Pentanone	8.20	96-22-0	979	980	$C_5H_{10}O$	MS+RI	Ether
	2	2,3-Pentanedione	10.34	600-14-6	1055	1056	$C_5H_8O_2$	MS+RI+STD	Buttery, caramellic, nutty, sweet, creamy, chessy
	3	2,6-Dimethyl-4-heptanone	14.38	108-83-8	1170	1178	$C_9H_{18}O$	MS+RI+STD	Green
	4	2-Hydroxy-3-pentanone	22.36	5704-20-1	1362	1364	$C_5H_{10}O_2$	MS+RI	Truffle, earth, nutty
	5	1-Hydroxy-2-butanone	23.11	5077-67-8	1379	1380	$C_4H_8O_2$	MS+RI	Sweet, coffee-like, moldy
	6	3-Ethyl-2-hydroxy-2-cyclopenten-1-one	36.40	21835-01-8	1694	1103	$C_7H_{10}O_2$	MS+RI	Maple-like, caramel-like, smoky, coffee-like, dairy, fruity
	7	1-(6-Methyl-2-pyrazinyl)-1-ethanone	36.81	22047-26-3	1706	1705	$C_7H_8N_2O$	MS+RI	Roasted coffee, cocoa-like, popcorn
	8	Methyl cyclopentenolone	40.56	80-71-7	1843	1034	$C_6H_8O_2$	MS+RI	Caramel-like, maple syrup
Alcohols	1	3-Methyl-1-butanol	15.58	123-51-3	1201	1205	$C_5H_{12}O$	MS+RI+STD	Whiskey, malt, burnt
	2	3-Methyl-1-pentanol	21.74	589-35-5	1348	1344	$C_6H_{14}O$	MS+RI+STD	Fruity, wine
	3	Furfuryl alcohol	35.34	98-00-0	1668	1670	$C_5H_6O_2$	MS+RI+STD	Burnt, sweet, bready, caramel-like
	4	Phenylethyl Alcohol	41.88	60-12-8	1919	1916	$C_8H_{10}O$	MS+RI+STD	Honey, spice, rose, lilac
Furans	1	3-Methylfuran	6.14	930-27-8	865	858	C_5H_6O	MS+RI	/
	2	2,5-Dimethylfuran	7.58	625-86-5	950	951	C_6H_8O	MS+RI	Meaty, roasted beef
	3	2-Vinylfuran	10.80	1487-18-9	1070	1075	C_6H_6O	MS+RI	Nutty, roasted

	4	2-Pentylfuran	16.54	3777-69-3	1224	1221	$C_9H_{14}O$	MS+RI	Green bean, butter
	5	2-Acetylfuran	29.04	1192-62-7	1515	1518	$C_6H_6O_2$	MS+RI	Almond-like, nutty, cocoa-like
	6	2,2-Methylenebis-furan	33.41	1197-40-6	1620	1615	$C_9H_8O_2$	MS+RI	Roasted, burnt (strong)
	7	2-Acetyl-5-methylfuran	33.66	1193-79-9	1626	1622	$C_7H_8O_2$	MS+RI	Nutty, caramel-like, sweet
	8	2-Furfuryl-5-methylfuran	36.20	13678-51-8	1689	1682	$C_{10}H_{10}O_2$	MS+RI	/
Pyrazines	1	Pyrazine	16.09	290-37-9	1216	1214	$C_4H_4N_2$	MS+RI	Nutty, burnt
	2	2-Methylpyrazine	18.31	109-08-0	1269	1267	$C_5H_6N_2$	MS+RI	Nutty, coffee, cocoa-like
	3	2,5-Dimethylpyrazine	20.66	123-32-0	1324	1324	$C_6H_8N_2$	MS+RI	Cocoa-like, roasted nut, roasted beef, medicine-like
	4	2,6-Dimethylpyrazine	20.92	108-50-9	1330	1331	$C_6H_8N_2$	MS+RI	Cocoa-like, roasted nut, roasted beef
	5	Ethylpyrazine	21.21	13925-00-3	1336	1338	$C_6H_8N_2$	MS+RI	Nutty coffee, cocoa-like
	6	2,3-Dimethylpyrazine	21.72	5910-89-4	1349	1350	$C_6H_8N_2$	MS+RI	Nutty, coffee, cocoa-like
	7	2-Ethyl-6-methylpyrazine	23.47	13925-03-6	1388	1384	$C_7H_{10}N_2$	MS+RI	Caramel-like, nutty, roasted potato
	8	2-Ethyl-5-methylpyrazine	23.76	13360-64-0	1395	1396	$C_7H_{10}N_2$	MS+RI	Caramel-like, coffee-like, nutty, roasted
	9	2-Propylpyrazine	24.99	18138-03-9	1422	1425	$C_7H_{10}N_2$	MS+RI	Green, vegetable, nutty
	10	Trimethyl-pyrazine	25.66	14667-55-1	1438	1437	$C_7H_{10}N_2$	MS+RI	Roasted, potato-like, must
	11	3-Ethyl-2,5-dimethyl-pyrazine	26.12	13360-65-1	1448	1450	$C_8H_{12}N_2$	MS+RI	Roasted potato, cocoa-like, nutty
	12	2,6-Diethyl-pyrazine	26.86	13067-27-1	1465	1463	$C_8H_{12}N_2$	MS+RI	Nutty, hazelnut
	13	2,3-Diethyl-5-methylpyrazine	28.24	18138-04-0	1496	1493	$C_7H_8N_2$	MS+RI+STD	Roasted (beef), nutty, meaty, hazelnut-like
	14	Acetylpyrazine	34.17	22047-25-2	1639	1638	$C_6H_6N_2O$	MS+RI	Roasted
Pyrroles	1	1-Methylpyrrole	13.21	96-54-8	1139	1145	C_5H_7N	MS+RI	Roasted, woody, herbal
	2	Pyrrole	29.48	109-97-7	1526	1523	C_4H_5N	MS+RI	Nutty, sweet
	3	1-(2-Furanylmethyl)-1H-pyrrole	40.64	1438-94-4	1848	1850	C_9H_9NO	MS+RI	Coffee-like, plastic-like, green, waxy, vegetable-like
	4	2-Acetyl pyrrole	42.70	1072-83-9	1966	1969	C_6H_7NO	MS+RI	Nutty, musty
Pyridines	1	Pyridine	14.85	110-86-1	1187	1186	C_5H_5N	MS+RI+STD	Sour, smoky, warm,burnt, coffee, putrid, fishy, amine
	2	3-Picoline	19.44	108-99-6	1295	1292	C_6H_7N	MS+RI	Smelly
	3	3-Ethylpyridine	23.26	536-78-7	1383	1384	C_7H_9N	MS+RI+STD	Smoky, tobacco-like, leather-like
Phenols	1	Guaiacol	41.16	90-05-1	1878	1876	$C_7H_8O_2$	MS+RI+STD	Phenolic, woody
	2	Phenol	43.08	108-95-2	1988	1990	C_6H_6O	MS+RI+STD	Phenolic, sweet, medicine-like
	3	4-Ethyl-2-methoxyphenol	43.43	2785-89-9	2008	2011	$C_9H_{12}O_2$	MS+RI+STD	Spicy, smoky, herbal, soy sauce flavor

	4	p-Cresol	44.10	106-44-5	2046	1077	C_7H_8O	MS+RI+STD	Medicine-like, phenolic, smoky
	5	2-Methoxy-4-vinylphenol	45.59	7786-61-0	2132	2138	$C_9H_{10}O_2$	MS+RI+STD	Spicy, sweet, clove-like, peppery, woody, powdery
Esters	1	Methyl isovalerate	9.19	556-24-1	1018	1019	$C_6H_{12}O_2$	MS+RI	Fruity, herbal
	2	Furfuryl formate	28.61	13493-97-5	1505	1504	$C_6H_6O_3$	MS+RI	Ethereal
	3	Furfuryl acetate	30.30	623-17-6	1545	1542	$C_7H_8O_3$	MS+RI+STD	Fruity, sweet, bananalike
	4	Furfuryl propanoate	32.82	623-19-8	1605	1602	$C_8H_{10}O_3$	MS+RI	Spicy, sweet, fruity
	5	Butyrolactone	34.41	96-48-0	1645	1645	$C_4H_6O_2$	MS+RI+STD	Caramel-like, sweet
Acids	1	Acetic acid	26.69	64-19-7	1461	1458	$C_2H_4O_2$	MS+RI+STD	Sour
	2	3-Methyl-butanoic acid	35.80	503-74-2	1680	1675	$C_5H_{10}O_2$	MS+RI+STD	Pungent, soury
	3	3,3-Dimethylacrylic acid	39.99	541-47-9	1811	1804	$C_5H_8O_2$	MS+RI	Phenolic, aldehyde, dairy products
Others	1	3-Methylthiophene	11.40	616-44-4	1089	1099	C_5H_6S	MS+RI	Fatty, winey
	2	2-(Methoxymethyl)furan	17.11	13679-46-4	1238	1245	$C_6H_8O_2$	MS+RI	Coffee-like, roasted
	3	Styrene	17.91	100-42-5	1258	1257	C_8H_8	MS+RI+STD	Balsamic, gasoline
	4	4-Methylthiazole	18.96	693-95-8	1283	1284	C_4H_5NS	MS+RI	Nutty, vegetable-like, tomato-like
	5	Difuryl ether	42.83	4437-22-3	1973	1299	$C_{10}H_{10}O_3$	MS+RI	Coffee-like, aged, stale, cardboard-like

[1] Retention indices in INNOWAX column.

[2] Qualitative analysis of VOCs was conducted: MS, by comparison of the mass spectrum with those included in the NIST 20 (National Institute of Standards and Technology) library; STD, by comparison of the mass spectrum and retention time with those of standard compounds.

[3] The odor descriptions were mainly sourced from the open-access website: website: www.flavornet.org/flavornet.html.

Table S5 Quantitative results of VOCs for coffee beans at different roasting stages.

Categories	No.	Name	Quantitative ion (m/z)	S0 (μg/kg)	SS1 (μg/kg)1	S2S2 (μg/kg)	S3S3 (μg/kg)	S4S4 (μg/kg)	S5 (μg/kg)5	S6 (μg/kg)S5	S7 (μg/kg)S5	S8 (μg/kg)S5
Aldehydes	1	2-Methylbutyraldehyde	6.87	788.29 ± 166.55 f	5261.99 ± 284.32 ab	4729.90 ± 233.68 bc	3582.08 ± 213.73 d	3004.60 ± 245.45 e	4408.95 ± 89.34 c	4706.88 ± 194.71 bc	5542.48 ± 180.48 a	4688.74 ± 87.29 c
	2	Hexanal	11.01	/	285.70 ± 12.16 ab	290.58 ± 3.27 ab	194.13 ± 53.90 b	244.48 ± 69.67 b	282.63 ± 57.99 ab	278.70 ± 56.98 ab	290.22 ± 107.20 ab	403.05 ± 26.89 a
	3	Nonanal	23.86	31.71 ± 9.12 a	/	/	/	/	/	/	/	/
	4	Furfural	27.28	/	46031.30 ± 1264.09 f	116768.83 ± 1112.79 d	163616.37 ± 6116.95 c	287233.06 ± 8091.23 a	297062.32 ± 3648.81 a	183661.96 ± 9161.03 b	126455.17 ± 2934.54 d	60432.81 ± 393.13 e
	5	Benzaldehyde	29.90	/	/	/	/	/	96.33 ± 19.98 c	1482.22 ± 321.65 b	2662.80 ± 257.30 a	2978.37 ± 181.76 a
	6	5-Methyl furfural	31.97	/	5184.36 ± 128.49 f	10613.29 ± 336.47 e	17046.69 ± 925.25 d	45342.67 ± 1481.31 b	52259.16 ± 1699.48 a	50869.94 ± 2669.22 a	29774.61 ± 1315.76 c	10927.82 ± 365.74 e
	7	1-Methyl-2-formylpyrrole	34.00	/	98.26 ± 5.15 gh	189.99 ± 5.19 fg	273.24 ± 15.22 f	740.89 ± 42.29 e	1169.19 ± 44.94 c	1799.23 ± 94.52 a	1440.51 ± 67.86 b	990.81 ± 79.81 d
	8	Benzeneacetaldehyde	34.90	146.17 ± 13.05 e	138.05 ± 8.85 e	114.79 ± 7.98 ef	97.25 ± 4.22 f	234.67 ± 20.33 cd	270.19 ± 14.11 bc	307.43 ± 19.89 ab	319.81 ± 18.00 a	228.11 ± 12.00 d
	9	2-Thiophenecarboxaldehyde	36.96	/	/	83.41 ± 2.18 e	112.13 ± 13.41 d	193.80 ± 10.84 c	233.28 ± 7.28 b	289.86 ± 18.27 a	239.34 ± 14.15 b	136.05 ± 1.91 d
	10	4-Ethylbenzaldehyde	38.60	/	11.10 ± 3.44 cd	17.33 ± 5.40 bc	19.60 ± 6.19 bc	25.34 ± 1.94 b	17.81 ± 1.74 bc	18.01 ± 1.11 bc	20.51 ± 0.77 bc	45.41 ± 9.17 a
	11	1H-Pyrrole-2-carboxaldehyde	43.43	4.44 ± 0.83 f	81.05 ± 8.97 f	164.71 ± 11.07 ef	296.38 ± 46.87 de	787.33 ± 32.12 bc	964.47 ± 147.97 ab	1122.52 ± 116.12 a	687.80 ± 84.85 c	387.06 ± 36.88 d
Ketones	1	3-Pentanone	8.20	/	178.92 ± 13.42 d	228.14 ± 17.95 d	225.00 ± 16.49 d	336.45 ± 23.21 c	437.81 ± 6.49 b	485.37 ± 37.80 b	658.49 ± 33.89 a	669.97 ± 71.90 a
	2	2,3-Pentanedione	10.34	/	571.23 ± 4.83 cd	814.53 ± 8.39 bc	865.42 ± 71.28 bc	1097.79 ± 51.19 ab	1388.13 ± 79.11 a	898.07 ± 137.49 bc	1038.73 ± 308.42 b	315.29 ± 37.00 de

	3	2,6-Dimethyl-4-heptanone	14.38	142.36 ± 2.47 c	144.72 ± 2.89 c	138.92 ± 1.55 c	220.85 ± 3.34 ab	230.18 ± 14.79 a	161.34 ± 3.64 bc	145.70 ± 44.50 c	133.19 ± 26.67 c	119.32 ± 30.95 c
	4	2-Hydroxy-3-pentanone	22.36	/	42.46 ± 2.92 a	/	/	/	/	/	/	/
	5	1-Hydroxy-2-butanone	23.11	/	/	102.54 ± 8.43 c	170.04 ± 7.42 b	296.83 ± 17.87 a	322.13 ± 22.30 a	218.64 ± 17.83 b	175.66 ± 39.39 b	99.51 ± 27.73 c
	6	3-Ethyl-2-hydroxy-2-cyclopenten-1-one	36.40	/	/	19.49 ± 1.47 e	44.50 ± 3.84 de	75.79 ± 4.25 d	437.81 ± 6.49 a	405.26 ± 39.93 ab	333.80 ± 25.99 c	356.79 ± 23.73 bc
	7	1-(6-Methyl-2-pyrazinyl)-1-ethanone	36.81	/	52.92 ± 5.48 d	81.91 ± 13.72 cd	111.61 ± 22.44 bc	150.97 ± 2.98 a	124.22 ± 19.75 ab	148.52 ± 10.11 a	96.72 ± 10.17 bc	78.87 ± 9.73 cd
	8	Methyl cyclopentenolone	40.56	/	21.82 ± 4.28 e	43.98 ± 1.47 de	72.61 ± 5.34 d	155.22 ± 15.65 c	167.38 ± 22.14 c	164.64 ± 27.37 c	234.17 ± 28.85 b	315.85 ± 6.99 a
Alcohols	1	3-Methyl-1-butanol	15.58	317.64 ± 79.17 a	56.25 ± 12.65 bc	44.62 ± 1.63 c	43.37 ± 5.12 c	54.84 ± 2.66 bc	78.70 ± 4.45 bc	95.97 ± 4.94 bc	123.40 ± 6.75 b	118.59 ± 11.12 bc
	2	3-Methyl-1-pentanol	21.74	139.76 ± 21.64 a	41.47 ± 2.56 b	45.32 ± 1.20 b	43.15 ± 2.78 b	43.51 ± 2.89 b	47.76 ± 6.45 b	46.12 ± 1.31 b	38.39 ± 1.30 b	29.63 ± 1.49 b
	3	Furfuryl alcohol	35.34	43312.14 ± 242.32 g	188658.15 ± 9758.41 f	323825.96 ± 6954.69 e	468008.81 ± 32868.58 d	728505.45 ± 17252.13 c	831536.04 ± 64702.47 bc	1114984.44 ± 70528.30 a	1165024.22 ± 75012.03 a	937497.95 ± 24678.55 b
	4	Phenylethyl Alcohol	41.88	6154.69 ± 1823.68 a	3011.97 ± 161.10 b	3273.58 ± 162.96 b	4338.37 ± 670.92 ab	6175.34 ± 124.39 a	5930.98 ± 750.71 a	6412.70 ± 388.37 a	4676.16 ± 308.30 ab	4975.79 ± 482.68 ab
Furans	1	3-Methylfuran	6.14	85.31 ± 4.74 f	340.27 ± 12.09 f	497.55 ± 33.35 f	549.93 ± 49.53 f	1243.78 ± 107.55 e	2754.87 ± 152.59 d	5326.30 ± 154.34 c	9352.14 ± 206.34 b	14120.80 ± 584.89 a
	2	2,5-Dimethylfuran	7.58	/	/	/	/	/	282.63 ± 14.00 d	1264.76 ± 60.00 c	2089.87 ± 60.49 b	2858.97 ± 87.76 a
	3	2-Vinylfuran	10.80	/	228.50 ± 5.43 de	341.93 ± 21.98 cde	288.22 ± 14.75 de	634.87 ± 106.39 cd	965.52 ± 222.46 bc	1536.90 ± 358.48 ab	2090.23 ± 334.47 a	2069.83 ± 358.17 a
	4	2-Pentylfuran	16.54	11.47 ± 0.89 d	117.55 ± 1.71 c	132.49 ± 2.32 c	127.61 ± 8.48 c	170.35 ± 8.17 b	216.56 ± 7.38 a	196.02 ± 7.22 a	194.31 ± 17.00 a	201.66 ± 6.68 a

	5	2-Acetylfuran	29.04	8.66 ± 0.47 h	414.38 ± 19.24 h	1061.21 ± 28.21 g	1718.06 ± 81.13 f	3859.26 ± 165.72 e	4615.68 ± 151.10 d	6512.45 ± 358.84 a	6005.90 ± 262.98 b	5282.54 ± 157.21 c
	6	2,2-Methylenebis-furan	33.41	/	/	8.92 ± 0.55 f	21.38 ± 1.75 f	147.71 ± 8.05 e	340.42 ± 9.67 d	934.78 ± 21.43 c	1656.23 ± 78.50 b	2138.80 ± 52.70 a
	7	2-Acetyl-5-methylfuran	33.66	/	/	/	/	128.18 ± 15.72 c	189.76 ± 11.10 b	373.43 ± 21.88 a	344.63 ± 17.54 a	379.33 ± 20.70 a
	8	2-Furfuryl-5-methylfuran	36.20	/	/	/	7.77 ± 0.59 ef	47.30 ± 1.71 e	110.20 ± 3.45 d	317.68 ± 3.49 c	586.87 ± 40.15 b	885.41 ± 17.03 a
Pyrazines	1	Pyrazine	16.09	60.25 ± 0.00 f	1723.33 ± 34.28 e	2066.54 ± 92.15 de	1960.66 ± 249.41 de	2204.57 ± 203.40 de	2353.21 ± 103.29 cd	2797.32 ± 143.68 c	4789.04 ± 418.97 a	4223.00 ± 60.92 b
	2	2-Methylpyrazine	18.31	51.94 ± 1.25 e	5510.12 ± 231.56 d	6938.36 ± 142.82 c	7029.52 ± 197.62 c	8157.22 ± 263.05 b	8184.42 ± 208.52 b	10033.53 ± 563.77 a	10109.80 ± 218.53 a	7891.01 ± 69.74 b
	3	2,5-Dimethylpyrazine	20.66	22.69 ± 1.29 e	3054.03 ± 134.02 d	3576.83 ± 135.15 cd	3692.78 ± 256.43 c	4398.78 ± 156.65 b	4042.88 ± 160.24 bc	5280.06 ± 341.06 a	4241.00 ± 147.35 b	3098.24 ± 123.52 d
	4	2,6-Dimethylpyrazine	20.92	17.95 ± 1.21 f	1986.00 ± 100.17 e	2638.74 ± 106.18 d	2884.78 ± 227.40 d	3910.50 ± 156.79 c	3832.06 ± 165.65 c	5287.35 ± 351.56 a	4444.87 ± 166.11 b	3583.44 ± 150.49 c
	5	Ethylpyrazine	21.21	9.63 ± 0.23 e	1191.40 ± 45.38 d	1681.02 ± 45.13 c	1785.28 ± 56.95 c	2498.71 ± 94.28 b	2652.53 ± 74.37 b	3467.88 ± 183.52 a	3291.46 ± 114.37 a	2646.03 ± 74.61 b
	6	2,3-Dimethylpyrazine	21.72	/	1470.79 ± 72.59 c	1893.93 ± 71.46 bc	2081.12 ± 153.62 bc	2845.59 ± 105.73 b	2842.44 ± 144.45 b	4533.15 ± 288.30 a	4284.20 ± 580.69 a	4692.37 ± 754.43 a
	7	2-Ethyl-6-methylpyrazine	23.47	/	931.79 ± 46.16 e	1352.82 ± 55.48 d	1519.22 ± 95.96 d	2185.73 ± 91.10 c	2293.75 ± 87.61 c	3167.21 ± 170.59 a	2722.15 ± 125.20 b	2193.29 ± 86.60 c
	8	2-Ethyl-5-methylpyrazine	23.76	5.36 ± 0.24 f	4576.90 ± 194.23 e	5622.93 ± 251.81 d	6131.08 ± 700.88 cd	7242.55 ± 270.54 b	7113.24 ± 357.08 bc	9225.97 ± 439.64 a	7574.52 ± 330.59 b	5459.58 ± 211.04 de
	9	2-Propylpyrazine	24.99	/	/	/	/	95.76 ± 3.34 c	122.74 ± 1.24 c	217.88 ± 8.25 b	277.75 ± 10.91 b	1399.65 ± 83.07 a
	10	Trimethyl-pyrazine	25.66	/	432.33 ± 26.26 c	639.14 ± 9.35 b	692.20 ± 21.41 b	1018.72 ± 52.84 a	/	/	/	/

	11	3-Ethyl-2,5-dimethyl-pyrazine	26.12	/	1965.24 ± 99.17 f	2476.24 ± 94.65 e	2640.46 ± 156.82 de	3255.19 ± 121.18 bc	3118.75 ± 134.05 bc	4072.20 ± 250.66 a	3388.47 ± 183.15 b	2889.24 ± 112.30 cd
	12	2,6-Diethyl-pyrazine	26.86	/	823.02 ± 45.94 e	1025.82 ± 9.92 de	1160.99 ± 32.21 d	1508.01 ± 83.05 c	1534.20 ± 51.13 c	2166.37 ± 136.01 a	1799.22 ± 144.47 b	1509.38 ± 93.45 c
	13	2,3-Diethyl-5-methylpyrazine	28.24	/	323.93 ± 12.75 e	391.60 ± 14.15 de	379.28 ± 12.42 de	457.71 ± 54.35 cd	486.43 ± 7.82 bc	584.20 ± 57.39 a	552.74 ± 26.92 ab	485.49 ± 10.27 bc
	14	Acetylpyrazine	34.17	/	30.43 ± 2.12 e	44.43 ± 3.73 de	72.82 ± 13.20 abc	90.48 ± 4.71 a	65.34 ± 14.08 bcd	87.22 ± 8.81 ab	58.85 ± 4.34 cd	45.83 ± 4.25 de
Pyrroles	1	1-Methylpyrrole	13.21	/	261.75 ± 16.41 e	320.00 ± 20.01 e	205.46 ± 5.75 ef	251.84 ± 8.48 e	713.54 ± 42.46 d	1229.45 ± 38.37 c	2918.69 ± 127.58 b	4529.82 ± 181.54 a
	2	Pyrrole	29.48	/	169.75 ± 13.45 cd	166.19 ± 21.35 cd	54.99 ± 10.03 d	54.43 ± 0.95 d	194.96 ± 26.46 cd	366.70 ± 15.00 c	1376.39 ± 76.71 b	2253.11 ± 197.22 a
	3	1-(2-Furanylmethyl)-1H-pyrrole	40.64	/	48.13 ± 3.11 gh	110.07 ± 3.76 fg	161.94 ± 7.00 f	468.17 ± 29.73 e	705.85 ± 15.98 d	1118.59 ± 32.13 c	1405.89 ± 88.58 b	1551.46 ± 23.45 a
	4	2-Acetyl pyrrole	42.70	3.76 ± 0.44 d	71.08 ± 9.31 cd	123.80 ± 11.27 cd	212.55 ± 39.52 c	435.35 ± 13.74 b	507.03 ± 86.72 b	916.21 ± 93.74 a	804.41 ± 71.61 a	882.56 ± 116.99 a
Pyridines	1	Pyridine	14.85	30.08 ± 2.22 f	473.32 ± 13.81 ef	635.58 ± 35.98 ef	787.44 ± 53.52 ef	1586.21 ± 56.41 e	3864.14 ± 40.70 d	9585.05 ± 392.49 c	21195.80 ± 228.51 b	43366.88 ± 1080.47 a
	2	3-Picoline	19.44	/	/	/	/	15.85 ± 1.82 de	45.73 ± 3.84 d	147.95 ± 14.17 c	400.54 ± 14.34 b	1444.48 ± 26.12 a
	3	3-Ethylpyridine	23.26	/	/	/	/	/	66.44 ± 15.75 d	265.68 ± 24.02 c	590.39 ± 35.75 b	1915.35 ± 22.80 a
Phenols	1	Guaiacol	41.16	/	11.52 ± 1.18 e	23.36 ± 1.44 e	41.30 ± 6.00 e	138.15 ± 4.87 de	230.10 ± 12.02 d	743.62 ± 49.36 c	989.59 ± 73.54 b	2092.79 ± 135.96 a
	2	Phenol	43.08	/	/	/	/	2189.88 ± 573.03 d	4096.95 ± 681.99 d	17259.50 ± 1269.36 c	25690.87 ± 2010.59 b	76428.73 ± 6457.04 a
	3	4-Ethyl-2-methoxyphenol	43.43	/	/	6.75 ± 0.72 d	14.23 ± 1.41 d	53.56 ± 2.16 d	85.45 ± 6.73 d	301.70 ± 18.69 c	483.91 ± 45.87 b	1186.53 ± 77.77 a

	4	p-Cresol	44.10	/	/	/	/	55.46 ± 5.88 c	107.14 ± 17.38 a	106.03 ± 10.61 ab	108.77 ± 14.50 a	79.55 ± 12.07 bc
	5	2-Methoxy-4-vinylphenol	45.59	/	182.83 ± 9.96 e	265.79 ± 20.50 de	336.63 ± 45.16 cd	455.16 ± 11.74 a	431.03 ± 58.60 abc	438.34 ± 54.19 ab	482.09 ± 41.27 a	344.51 ± 18.62 bcd
Esters	1	Methyl isovalerate	9.19	340.55 ± 4.84 a	/	/	/	/	/	/	/	/
	2	Furfuryl formate	28.61	/	33.49 ± 1.32 f	2670.91 ± 89.59 e	2705.62 ± 206.38 e	5713.95 ± 251.68 d	14156.47 ± 986.80 c	15966.72 ± 1348.45 b	31626.68 ± 554.98 a	13764.54 ± 550.74 c
	3	Furfuryl acetate	30.30	/	2331.37 ± 51.20 ef	4634.81 ± 91.67 ef	7707.70 ± 234.84 e	28041.96 ± 1258.22 d	48530.22 ± 792.99 c	92975.81 ± 3406.34 b	119436.68 ± 5125.15 a	95895.75 ± 1007.38 b
	4	Furfuryl propanoate	32.82	/	96.59 ± 5.45 f	316.18 ± 6.79 f	611.98 ± 22.74 f	2875.48 ± 84.71 e	4903.99 ± 101.80 d	11160.41 ± 225.53 c	18754.62 ± 826.31 b	23834.38 ± 554.80 a
	5	Butyrolactone	34.41	/	47.73 ± 4.26 ef	62.31 ± 4.27 ef	93.28 ± 2.93 e	180.01 ± 4.24 d	239.19 ± 32.62 d	376.84 ± 49.62 c	658.05 ± 54.48 b	980.43 ± 9.49 a
Acids	1	Acetic acid	26.69	/	192.85 ± 27.26 de	360.02 ± 54.10 cd	646.87 ± 90.26 b	878.67 ± 71.07 a	901.76 ± 106.90 a	461.03 ± 104.27 bc	339.03 ± 65.52 cd	64.69 ± 4.57 e
	2	3-Methyl-butanoic acid	35.80	106.15 ± 26.76 e	751.68 ± 57.96 d	1158.58 ± 197.76 bc	1405.84 ± 177.45 ab	1800.80 ± 95.91 a	1726.87 ± 232.95 a	1641.87 ± 182.82 a	807.64 ± 81.81 cd	320.46 ± 26.08 e
	3	3,3-Dimethylacrylic acid	39.99	/	129.45 ± 13.41 de	182.21 ± 40.69 cd	259.85 ± 44.94 abc	305.07 ± 16.89 a	260.98 ± 42.81 ab	185.45 ± 24.63 bcd	81.01 ± 6.79 ef	20.79 ± 1.87 fg
Others	1	3-Methylthiophene	11.40	/	/	79.46 ± 1.92 f	85.51 ± 2.29 f	153.90 ± 5.22 e	293.41 ± 14.61 d	516.99 ± 8.57 c	814.36 ± 46.55 b	1187.37 ± 18.86 a
	2	2-(Methoxymethyl)furan	17.11	/	/	35.38 ± 1.53 e	64.84 ± 2.23 e	227.28 ± 3.20 d	495.45 ± 2.81 c	977.17 ± 41.75 b	1237.16 ± 58.62 a	938.51 ± 2.86 b
	3	Styrene	17.91	36.38 ± 2.66 g	132.57 ± 2.31 f	170.18 ± 1.19 e	151.54 ± 5.53 ef	176.02 ± 10.95 de	200.71 ± 3.59 d	274.79 ± 13.92 c	377.02 ± 17.57 b	808.03 ± 15.48 a
	4	4-Methylthiazole	18.96	/	64.13 ± 1.54 g	112.58 ± 2.67 f	122.02 ± 6.34 f	178.63 ± 4.05 e	212.49 ± 5.28 d	261.03 ± 12.30 c	334.11 ± 8.51 a	304.00 ± 11.95 b

5	Difuryl ether	42.83	/	/	/	/	51.89 ± 4.83 d	87.81 ± 4.76 d	262.06 ± 20.27 c	352.92 ± 26.65 b	425.75 ± 26.05 a

Values are represented as mean ± SD (n=3);

"/" indicates not detected;

"a-g" indicates a significant difference ($p \leq 0.05$) for different lowercase letters in the same row;

“S0-S8” indicates different roasting stages.

Table S6 OAVs of VOCs for coffee beans at different roasting stages.

Categories	No.	Name	Threshold (mg/kg)[1]	S0	S1	S2	S3	S4	S5	S6	S7	S8
Aldehydes	1	2-Methylbutyraldehyde	0.001	788.28871	5261.99093	4729.89480	3582.07593	3004.60240	4408.95455	4706.87603	5542.47830	4688.73627
	2	Hexanal	0.005	0.00000	57.14011	58.11606	38.82669	48.89564	56.52614	55.74031	58.04352	80.61087
	3	Nonanal	0.0044	7.20638	0.00000	0.00000	0.00000	0.00000	0.00000	0.00000	0.00000	0.00000
	4	Furfural	9.562	0.00000	4.81398	12.21176	17.11110	30.03901	31.06697	19.20748	13.22476	6.32010
	5	Benzaldehyde	0.75089	0.00000	0.00000	0.00000	0.00000	0.00000	0.12828	1.97395	3.54619	3.96645
	6	5-Methyl furfural	1.11	0.00000	4.67060	9.56152	15.35738	40.84925	47.08032	45.82878	26.82397	9.84488
	7	1-Methyl-2-formylpyrrole	0.037	0.00000	2.65559	5.13476	7.38495	20.02407	31.59958	48.62779	38.93276	26.77865
	8	Benzeneacetaldehyde	0.0063	23.20105	21.91259	18.22016	15.43732	37.25009	42.88722	48.79847	50.76289	36.20830
	9	2-Thiophenecarboxaldehyde	5	0.00000	0.00000	0.01668	0.02243	0.03876	0.04666	0.05797	0.04787	0.02721
	10	4-Ethylbenzaldehyde	/	/	/	/	/	/	/	/	/	/
	11	1H-Pyrrole-2-carboxaldehyde	65	0.00007	0.00125	0.00253	0.00456	0.01211	0.01484	0.01727	0.01058	0.00595
Ketones	1	3-Pentanone	0.061	0.00000	2.93305	3.74005	3.68857	5.51557	7.17724	7.95691	10.79500	10.98314
	2	2,3-Pentanedione	0.03	0.00000	19.04117	27.15087	28.84736	36.59282	46.27104	29.93570	34.62442	10.50955
	3	2,6-Dimethyl-4-heptanone	/	/	/	/	/	/	/	/	/	/
	4	2-Hydroxy-3-pentanone	/	/	/	/	/	/	/	/	/	/
	5	1-Hydroxy-2-butanone	/	/	/	/	/	/	/	/	/	/
	6	3-Ethyl-2-hydroxy-2-cyclopenten-1-one	0.3	0.00000	0.00000	0.06498	0.14834	0.25264	1.45937	1.35085	1.11266	1.18930
	7	1-(6-Methyl-2-pyrazinyl)-1-ethanone	0.3	0.00000	0.17641	0.27304	0.37203	0.50322	0.41406	0.49507	0.32240	0.26290
	8	Methyl cyclopentenolone	0.3	0.00000	0.07273	0.14659	0.24202	0.51740	0.55793	0.54879	0.78057	1.05282
Alcohols	1	3-Methyl-1-butanol	0.004	79.41124	14.06230	11.15395	10.84167	13.71015	19.67635	23.99167	30.84993	29.64759
	2	3-Methyl-1-pentanol	0.0075	18.63470	5.52977	6.04255	5.75332	5.80112	6.36780	6.14938	5.11911	3.95097
	3	Furfuryl alcohol	4.5005	9.62385	41.91938	71.95333	103.99040	161.87212	184.76526	247.74679	258.86551	208.30973
	4	Phenylethyl Alcohol	1	6.15469	3.01197	3.27358	4.33837	6.17535	5.93098	6.41270	4.67616	4.97579
Furans	1	3-Methylfuran	/	/	/	/	/	/	/	/	/	/
	2	2,5-Dimethylfuran	1	0.00000	0.00000	0.00000	0.00000	0.00000	0.28263	1.26476	2.08987	2.85898
	3	2-Vinylfuran	1	0.00000	0.22850	0.34192	0.28822	0.63487	0.96552	1.53690	2.09023	2.06984

	4	2-Pentylfuran	0.006	1.91270	19.59194	22.08073	21.26861	28.39214	36.09272	32.66972	32.38527	33.61108
	5	2-Acetylfuran	15.0252	0.00058	0.02758	0.07063	0.11435	0.25685	0.30720	0.43344	0.39972	0.35158
	6	2,2-Methylenebis-furan	1	0.00000	0.00000	0.00892	0.02138	0.14771	0.34042	0.93478	1.65623	2.13881
	7	2-Acetyl-5-methylfuran	0.0002	0.00000	0.00000	0.00000	0.00000	640.92245	948.77456	1867.12940	1723.14383	1896.63345
	8	2-Furfuryl-5-methylfuran	/	/	/	/	/	/	/	/	/	/
Pyrazines	1	Pyrazine	300	0.00020	0.00574	0.00689	0.00654	0.00735	0.00784	0.00932	0.01596	0.01408
	2	2-Methylpyrazine	30	0.00173	0.18367	0.23128	0.23432	0.27191	0.27281	0.33445	0.33699	0.26303
	3	2,5-Dimethylpyrazine	1.75	0.01297	1.74516	2.04390	2.11016	2.51359	2.31022	3.01717	2.42343	1.77042
	4	2,6-Dimethylpyrazine	0.718	0.02500	2.76601	3.67513	4.01779	5.44637	5.33714	7.36399	6.19063	4.99087
	5	Ethylpyrazine	4	0.00241	0.29785	0.42025	0.44632	0.62468	0.66313	0.86697	0.82286	0.66151
	6	2,3-Dimethylpyrazine	0.8	0.00000	1.83849	2.36742	2.60140	3.55699	3.55305	5.66644	5.35525	5.86546
	7	2-Ethyl-6-methylpyrazine	0.04	0.00000	23.29468	33.82055	37.98047	54.64334	57.34373	79.18032	68.05368	54.83222
	8	2-Ethyl-5-methylpyrazine	0.016	0.33476	286.05612	351.43296	383.19272	452.65940	444.57737	576.62330	473.40764	341.22413
	9	2-Propylpyrazine	0.3	0.00000	0.00000	0.00000	0.00000	0.31921	0.40913	0.72629	0.92584	4.66549
	10	Trimethyl-pyrazine	0.35012	0.00000	1.23481	1.82551	1.97704	2.90962	0.00000	0.00000	0.00000	0.00000
	11	3-Ethyl-2,5-dimethyl-pyrazine	0.0086	0.00000	228.51635	287.93475	307.02980	378.51057	362.64531	473.51136	394.00834	335.95791
	12	2,6-Diethyl-pyrazine	0.006	0.00000	137.16975	170.97030	193.49864	251.33425	255.70008	361.06131	299.86936	251.56322
	13	2,3-Diethyl-5-methylpyrazine	0.000084	0.00000	3856.32781	4661.91210	4515.22300	5448.94210	5790.77989	6954.78835	6580.28642	5779.65341
	14	Acetylpyrazine	0.061	0.00000	0.49885	0.72841	1.19380	1.48330	1.07123	1.42982	0.96474	0.75131
Pyrroles	1	1-Methylpyrrole	>0.037	0.00000	7.07434	8.64870	5.55305	6.80649	19.28492	33.22837	78.88351	122.42756
	2	Pyrrole	20	0.00000	0.00849	0.00831	0.00275	0.00272	0.00975	0.01833	0.06882	0.11266
	3	1-(2-Furanylmethyl)-1H-pyrrole	0.1	0.00000	0.48128	1.10072	1.61939	4.68166	7.05847	11.18587	14.05885	15.51457
	4	2-Acetyl pyrrole	58.58525	0.00006	0.00121	0.00211	0.00363	0.00743	0.00865	0.01564	0.01373	0.01506
Pyridines	1	Pyridine	2	0.01504	0.23666	0.31779	0.39372	0.79311	1.93207	4.79253	10.59790	21.68344
	2	3-Picoline	/	/	/	/	/	/	/	/	/	/
	3	3-Ethylpyridine	>0.019	0.00000	0.00000	0.00000	0.00000	0.00000	3.49699	13.98336	31.07335	100.80766
Phenols	1	Guaiacol	0.0016	0.00000	7.20153	14.59678	25.80897	86.34346	143.81233	464.76133	618.49520	1307.99185
	2	Phenol	58.58525	0.00000	0.00000	0.00000	0.00000	0.03738	0.06993	0.29460	0.43852	1.30457
	3	4-Ethyl-2-methoxyphenol	0.08925	0.00000	0.00000	0.07567	0.15945	0.60008	0.95745	3.38037	5.42193	13.29443

	4	p-Cresol	0.055	0.00000	0.00000	0.00000	0.00000	1.00847	1.94806	1.92785	1.97772	1.44646
	5	2-Methoxy-4-vinylphenol	0.01202	0.00000	15.21077	22.11259	28.00636	37.86667	35.85925	36.46777	40.10696	28.66117
Esters	1	Methyl isovalerate	0.044	7.73985	0.00000	0.00000	0.00000	0.00000	0.00000	0.00000	0.00000	0.00000
	2	Furfuryl formate	/	/	/	/	/	/	/	/	/	/
	3	Furfuryl acetate	/	/	/	/	/	/	/	/	/	/
	4	Furfuryl propanoate	/	/	/	/	/	/	/	/	/	/
	5	Butyrolactone	35	0.00000	0.00136	0.00178	0.00267	0.00514	0.00683	0.01077	0.01880	0.02801
Acids	1	Acetic acid	22	0.00000	0.00877	0.01636	0.02940	0.03994	0.04099	0.02096	0.01541	0.00294
	2	3-Methyl-butanoic acid	0.49	0.21663	1.53403	2.36445	2.86907	3.67511	3.52423	3.35075	1.64824	0.65400
	3	3,3-Dimethylacrylic acid	14	0.00000	0.00925	0.01302	0.01856	0.02179	0.01864	0.01325	0.00579	0.00149
Others	1	3-Methylthiophene	/	/	/	/	/	/	/	/	/	/
	2	2-(Methoxymethyl)furan	>27	0.00000	0.00000	0.00131	0.00240	0.00842	0.01835	0.03619	0.04582	0.03476
	3	Styrene	0.05	0.72768	2.65137	3.40368	3.03074	3.52041	4.01427	5.49582	7.54031	16.16052
	4	4-Methylthiazole	0.055	0.00000	1.16593	2.04688	2.21864	3.24787	3.86352	4.74600	6.07462	5.52728
	5	Difuryl ether	/	/	/	/	/	/	/	/	/	/

[1]The odour thresholds were mainly collected from a book: Compilations of odour threshold values in air, water, and other media (second enlarged and revised edition).
